# Supplementary material for: Consumer understanding of terms used in imaging reports requested for low back pain: a cross-sectional survey
Source: BMJ Open. 2021 Sep 9;11(9):e049938. doi: 10.1136/bmjopen-2021-049938 (PMC8438839; doi:10.1136/bmjopen-2021-049938)
Supplement: Supplementary data [file bmjopen-2021-049938supp001.pdf]

Supplementary File

Note that for all questions the current analysis is dichotomised into strongly disagree/somewhat disagree as one value and Neutral/Somewhat agree/Strongly agree as the other.

Sensitivity analysis A: Questions regarding seriousness, persistence and fear of movement dichotomised into Strongly disagree/Somewhat disagree/neutral compared with Somewhat agree/Strongly agree. Comprehension remains unchanged.

Table 1: ***Association between the self-reported understanding of the 14 terms overall and perceived seriousness, pain persistence and fear of movement, Spearman's rho correlation****

	**Understanding**	**Seriousness**	**Pain persistence**	**Fear of movement**
**Understanding**	1			
**Seriousness**	0.29	1		
**Persistence**	0.21	0.72	1	
**Fear of movement**	0.19	0.53	0.6	1

All p values <0.001.

Values between 0.10 and 0.29 represent a weak association, 0.30-0.49 moderate and 0.5 and above a strong association.

Table 2: Association between self-reported understanding of the 14 terms, perceived seriousness, pain persistence and fear of movement and demographic details, history of back pain and back beliefs

	**Understanding**	**Seriousness**	**Pain persistence**	**Fear of movement**
	Regression coefficient (95% Confidence Interval)			
**Education (REF - High school)**				
**Trade/Diploma**	0.36 (-0.01, 0.73)	0.19 (-0.17, 0.56)	-0.07 (-0.42, 0.29)	-0.12 (-0.49, 0.24)
**Bachelor**	**0.40 (0.07, 0.72)***	0.02 (-0.28, 0.32)	0.04 (-0.26, 0.34)	-0.09 (-0.39, 0.22)
**Postgraduate qualifications**	**0.66 (0.23, 1.08)****	-0.07 (-0.46, 0.33)	-0.15 (-0.53, 0.23)	0.03 (-0.37, 0.43)
**LBP History (REF = Never)**				
**Previous LBP**	0.27 (-0.21, 0.75)	0.12 (-0.28, 0.52)	0.15 (-0.23, 0.53)	-0.06 (-0.46, 0.36)
**Current LBP**	**0.50 (0.02, 0.97)***	0.06 (-0.34, 0.47)	0.14 (-0.25, 0.52)	**-0.60 (-1.00, -0.19)****
**BBQ score**	**0.02 (0.0003, 0.04)***	**-0.02 (-0.04, -0.005)***	**-0.05 (-0.07, -0.03)*****	**-0.07 (-0.09, -0.05)*****
**Age, years**	0.002 (-0.007, 0.01)	0.002 (-0.01, 0.01)	0.003 (-0.004, 0.011)	**-0.01 (-0.02, -0.01)****
**Gender (REF = Female)**				
**Male**	0.07 (-0.20, 0.33)	**-0.39 (-0.64, -0.14)****	**-0.32 (-0.57, -0.08)***	-0.24 (-0.49, 0.01)

Note: Bold indicates statistically significant, * p < 0.05, **p < 0.01, ***p < 0.001. Results rounded to two decimal points or first meaningful decimal point. REF = Reference category, LBP = Low Back Pain, BBQ = Back Beliefs Questionnaire

Continuous variables (BBQ score and age): For understanding, a positive regression coefficient indicates greater self-reported understanding is associated with better back beliefs (higher BBQ score) and increased age. For seriousness, pain persistence and fear of movement, a positive regression coefficient indicates greater worry/concern is associated with poorer back beliefs (lower BBQ score) and increased age.

Categorical variables: For understanding, a positive regression coefficient indicates increased self-reported understanding compared with the reference category. For seriousness, pain persistence and fear of movement, a positive regression coefficient indicates greater worry/concern compared with the reference variable.

Sensitivity analysis B: Neutral answers removed. Dichotomy is strongly disagree/somewhat disagree vs somewhat agree/strongly agree.

Table 3: ***Association between the self-reported understanding of the 14 terms overall and perceived seriousness, pain persistence and fear of movement, Spearman's rho correlation****

	Understanding	Seriousness	Pain persistence	Fear of movement
**Understanding**	1			
**Seriousness**	0.60	1		
**Persistence**	0.45	0.72	1	
**Fear of movement**	0.37	0.53	0.6	1

******All p values <0.001.*

Values between 0.10 and 0.29 represent a weak association, 0.30-0.49 moderate and 0.5 and above a strong association.

Table 4: Association between self-reported understanding of the 14 terms, perceived seriousness, pain persistence and fear of movement and demographic details, history of back pain and back beliefs

	**Understanding**	**Seriousness**	**Pain persistence**	**Fear of movement**
	Regression coefficient (95% Confidence Interval)			
Education (REF - High school)				
Trade/Diploma	0.27 (-0.11, 0.65)	0.005 (-0.59, 0.60)	-0.42 (-0.97, 0.13)	-0.39 (-0.88, 0.09)
Bachelor	0.32 (-0.01, 0.66)	- 0.16 (-0.63, 0.31)	-0.19 (-0.63, 0.26)	**-0.45 (-0.87, -0.04)***
Postgraduate qualifications	**0.55 (0.12, 0.98)***	0.43 (-1.03, 0.17)	**-0.77 (-1.32, -0.23)****	**-0.65 (-1.15, -0.14)***
LBP History (REF = Never)				
Previous LBP	0.18 (-0.30, 0.66)	0.21 (-0.41, 0.83)	-0.01 (-0.58, 0.56)	-0.19 (-0.71, 0.33)
Current LBP	**0.55 (0.07, 1.02)***	-0.01 (-0.63, 0.61)	-0.18 (-0.76, 0.40)	**-0.61 (-1.14, -0.08)***
BBQ score	**0.03 (0.01, 0.05)***	**-0.04 (-0.07, -0.01)****	**-0.08 (-0.11, -0.05)*****	**-0.08 (-0.11, -0.05)*****
Age, years	-0.003 (-0.01, 0.01)	**0.02 (0.01, 0.04)*****	**0.03 (0.02, 0.04)*****	-0.01 (-0.02, 0.001)
Gender (REF = Female)				
Male	0.17 (-0.10, 0.44)	**-0.49 (-0.90, -0.08)***	-0.27 (-0.64, 0.11)	-0.30 (-0.64, 0.04)

Note: Bold indicates statistically significant, * p < 0.05, **p < 0.01, ***p < 0.001. Results rounded to two decimal points or first meaningful decimal point. REF = Reference category, LBP = Low Back Pain, BBQ = Back Beliefs Questionnaire

Continuous variables (BBQ score and age): For understanding, a positive regression coefficient indicates greater self-reported understanding is associated with better back beliefs (higher BBQ score) and increased age. For seriousness, pain persistence and fear of movement, a positive regression coefficient indicates greater worry/concern is associated with poorer back beliefs (lower BBQ score) and increased age.

Categorical variables: For understanding, a positive regression coefficient indicates increased self-reported understanding compared with the reference category. For seriousness, pain persistence and fear of movement, a positive regression coefficient indicates greater worry/concern compared with the reference variable.
