# Supplementary material for: Consumer understanding of terms used in imaging reports requested for low back pain: a cross-sectional survey
Source: BMJ Open. 2021 Sep 9;11(9):e049938. doi: 10.1136/bmjopen-2021-049938 (PMC8438839; doi:10.1136/bmjopen-2021-049938)
Supplement: Author's manuscript [file bmjopen-2021-049938.draft_revisions.pdf]

BMJ Open

BMJ Open is committed to open peer review. As part of this commitment we make the peer review history of every article we publish publicly available.

When an article is published we post the peer reviewers' comments and the authors' responses online. We also post the versions of the paper that were used during peer review. These are the versions that the peer review comments apply to.

The versions of the paper that follow are the versions that were submitted during the peer review process. They are not the versions of record or the final published versions. They should not be cited or distributed as the published version of this manuscript.

BMJ Open is an open access journal and the full, final, typeset and author-corrected version of record of the manuscript is available on our site with no access controls, subscription charges or pay-per-view fees (http://bmjopen.bmj.com).

If you have any questions on BMJ Open's open peer review process please email info.bmjopen@bmj.com

BMJ Open

Consumer understanding of terms used in imaging reports requested for low back pain

Journal:	*BMJ Open*
Manuscript ID	bmjopen-2021-049938
Article Type:	Original research
Date Submitted by the Author:	10-Feb-2021
Complete List of Authors:	Farmer, Caitlin; Monash University, Department of Epidemiology and Preventive Medicine, School of Public Health and Preventive Medicine; Cabrini Institute, Department of Clinical Epidemiology, Cabrini Institute<br>O'Connor, Denise; Monash University, Department of Epidemiology and Preventive Medicine, School of Public Health and Preventive Medicine; Monash University, Department of Clinical Epidemiology, Cabrini Institute<br>Lee, Hopin; University of Oxford, Centre for Statistics in Medicine, Rehabilitation Research in Oxford, Nuffield Department of Orthopaedics Rheumatology and Musculoskeletal Sciences (NDORMS), ; The University of Newcastle, School of Medicine and Public Health<br>McCaffery, Kirsten; The University of Sydney, Sydney Health Literacy Lab, School of Public Health<br>Maher, Christopher; The University of Sydney, School of Public Health; The University of Sydney Institute for Musculoskeletal Health<br>Newell, Dave; AECC University College<br>Cashin, Aidan; Neuroscience Research Australia, Centre for Pain IMPACT; University of New South Wales, Prince of Wales Clinical School<br>Byfield, David; University of South Wales Faculty of Life Sciences and Education<br>Jarvik, Jeffrey; University of Washington, Departments of Radiology, Neurological Surgery and Health Services, School of Medicine; University of Washington, UW Clinical Learning, Evidence And Research (CLEAR) Center for Musculoskeletal Disorders<br>Buchbinder, Rachelle; Monash University Faculty of Medicine Nursing and Health Sciences, Dept of Epidemiology and Preventive Medicine, School of Public Health and Preventive Medicine; Monash University, Department of Clinical Epidemiology, Cabrini Institute
Keywords:	Diagnostic radiology < RADIOLOGY & IMAGING, Back pain < ORTHOPAEDIC & TRAUMA SURGERY, Musculoskeletal disorders < ORTHOPAEDIC & TRAUMA SURGERY

SCHOLARONE™
Manuscripts

I, the Submitting Author has the right to grant and does grant on behalf of all authors of the Work (as defined in the below author licence), an exclusive licence and/or a non-exclusive licence for contributions from authors who are: i) UK Crown employees; ii) where BMJ has agreed a CC-BY licence shall apply, and/or iii) in accordance with the terms applicable for US Federal Government officers or employees acting as part of their official duties; on a worldwide, perpetual, irrevocable, royalty-free basis to BMJ Publishing Group Ltd ("BMJ") its licensees and where the relevant Journal is co-owned by BMJ to the co-owners of the Journal, to publish the Work in this journal and any other BMJ products and to exploit all rights, as set out in our licence.

The Submitting Author accepts and understands that any supply made under these terms is made by BMJ to the Submitting Author unless you are acting as an employee on behalf of your employer or a postgraduate student of an affiliated institution which is paying any applicable article publishing charge ("APC") for Open Access articles. Where the Submitting Author wishes to make the Work available on an Open Access basis (and intends to pay the relevant APC), the terms of reuse of such Open Access shall be governed by a Creative Commons licence – details of these licences and which Creative Commons licence will apply to this Work are set out in our licence referred to above.

Other than as permitted in any relevant BMJ Author's Self Archiving Policies, I confirm this Work has not been accepted for publication elsewhere, is not being considered for publication elsewhere and does not duplicate material already published. I confirm all authors consent to publication of this Work and authorise the granting of this licence.

For peer review only

Consumer understanding of terms used in imaging reports requested for low back pain

Caitlin Farmer[1,2], Denise O'Connor[1,2], Hopin Lee[3,4], Kirsten McCaffery[5], Christopher Maher[6,7], David Newell[8], Aidan Cashin[9,10], David Byfield[11], Jeffrey G. Jarvik[12,13], Rachelle Buchbinder[1,2]

[1]Department of Epidemiology and Preventive Medicine, School of Public Health and Preventive Medicine, Monash University, Melbourne, Australia.

[2]Monash Department of Clinical Epidemiology, Cabrini Institute, Melbourne, Australia.

[3]Centre for Statistics in Medicine, Rehabilitation Research in Oxford, Nuffield Department of Orthopaedics Rheumatology and Musculoskeletal Sciences (NDORMS), University of Oxford, Oxford, United Kingdom.

[4]School of Medicine and Public Health, University of Newcastle, Newcastle, Australia.

[5]Sydney Health Literacy Lab, Wiser Healthcare, Sydney School of Public Health, Faculty of Medicine and Health, University of Sydney, Sydney, Australia

[6]School of Public Health, University of Sydney, Sydney, Australia.

[7]Institute for Musculoskeletal Health, Sydney, Australia.

[8]AECC University College, Dorset, United Kingdom.

[9]Centre for Pain IMPACT, Neuroscience Research Australia, Sydney, Australia.

[10]Prince of Wales Clinical School, Faculty of Medicine, University of New South Wales, Sydney, Australia.

[11]Faculty of Life Sciences and Education University of South Wales, Treforest, United Kingdom.

[12]Departments of Radiology, Neurological Surgery and Health Services, School of Medicine, University of Washington, United States.

[13]UW Clinical Learning, Evidence And Research (CLEAR) Center for Musculoskeletal Disorders, Seattle, WA, United States.

Corresponding author:

Caitlin Farmer

Monash Department of Clinical Epidemiology

Cabrini Institute

4 Drysdale St

Malvern VIC 3144

Australia

caitlin.farmer1@monash.edu

Word Count: 2999 excluding title page, abstract, tables and references

For peer review only

For peer review only - http://bmjopen.bmj.com/site/about/guidelines.xhtml

ABSTRACT

Objectives: To investigate 1) self-reported societal comprehension of common and usually non-serious terms found in lumbar spine imaging reports and 2) its relationship to perceived seriousness, likely persistence of low back pain (LBP), fear of movement, back beliefs and history and intensity of low back pain.

Design: Cross-sectional online survey of the general public.

Setting: Five English-speaking countries: United Kingdom, United States, Canada, New Zealand, Australia.

Participants: Adults (age > 18 years) with or without a history of LBP recruited in April 2019 with quotas for country, age and gender.

Primary and secondary outcome measures: Self-reported understanding of 14 terms (annular fissure, disc bulge, disc degeneration, disc extrusion, disc height loss, disc protrusion, disc signal loss, facet joint degeneration, high intensity zone, mild canal stenosis, modic changes, nerve root contact, spondylolisthesis and spondylosis) commonly found in lumbar spine imaging reports. For each term we also elicited worry about its seriousness, and whether its presence would indicate pain persistence and prompt fear of movement.

Results: From 774 responses we included 677 (87.5%) with complete and valid responses. 577 (85%) participants had a current or past history of LBP of whom 251 (44%) had received lumbar spine imaging. Self-reported understanding of all terms was poor. At best, 235 (35%) reported understanding the term ‘disc degeneration’, while only 71 (10.5%) reported understanding the term ‘modic changes’. For all terms, a moderate to large proportion of participants (range 59-71%), considered they indicated a serious back problem, that pain might persist (range 52-71%) and they would be fearful of movement (range 42-57%).

Conclusion: Common and usually non-serious terms in lumbar spine imaging reports are poorly understood by the general population and may contribute to the burden of LBP.

Trial registration: This study was prospectively registered with the Australian & New Zealand Clinical Trials Registry (12619000545167).

STRENGTHS AND LIMITATIONS OF THIS STUDY

- This is the first study to directly measure societal self-reported understanding of 14 commonly used terms found in lumbar spine imaging reports and its with worry regarding the seriousness and persistence of low back pain and being fearful of movement.
- Participants were a large representative sample from five high-income English-speaking countries with quotas for age and gender suggesting our results are likely generalisable to those settings but may or may not be generalisable to other settings.
- Self-reported understanding may not truly reflect a person's understanding of the terms, and the use of a survey sampling company may have introduced some bias.
- Although all terms that we investigated are findings that commonly exist in the asymptomatic population, some are more common in people with low back pain and this may have influenced participants' responses.

For peer review only

For peer review only - http://bmjopen.bmj.com/site/about/guidelines.xhtml

INTRODUCTION

Low back pain (LBP) is a common, costly problem and a significant contributor to global disability.(1) Most LBP is labelled 'non-specific', reflecting an inability to identify a specific cause in most cases.(2) In the absence of features suggestive of a serious cause, clinical practice guidelines consistently recommend against any form of lumbar spine imaging as it is unlikely to provide meaningful information about the cause of pain or guide treatment.(3-5) Imaging often reveals age-related changes such as disc degeneration, disc bulge and facet joint degeneration that are also common in asymptomatic people.(6)

Lumbar spine imaging also carries risk of iatrogenic harm. This includes not only exposure to ionising radiation with plain radiographs and CT scans, but the way findings are described may lead to clinician and patient anxiety about perceived seriousness, prompt further unnecessary tests, unwarranted diagnostic labels and unnecessary treatment.(7, 8)

While few published studies have investigated consumer understanding and views regarding terminology in lumbar spine imaging findings in people with LBP, several studies have investigated these issues for other conditions.(9-14) A systematic review investigating how different terms for various conditions influence management preferences found that use of more medicalised or precise terms generally results in greater anxiety and perceived severity, and preferences towards more invasive care.(15) Ambiguous terminology for incidental low-risk liver lesions reported in abdominal CT reports was found to increase patient and referrer anxiety and a greater likelihood of follow-up testing.(16) In contrast, simplified upper limb MRI reports using more understandable and less 'emotive' language (e.g., 'expected age-related changes' rather than 'hypertrophic degenerative changes') have been found to reduce worry, improve comprehension and help patients feel more in control of their health.(17)

For peer review only

As patients are increasingly accessing their imaging reports, it is important to understand how commonly reported terms are interpreted by patients. The primary aim of this study was to investigate self-reported understanding of terms that are both commonly found in lumbar spine imaging reports and are usually of little clinical relevance. We also investigated the relationship between self-reported understanding of these terms and their perceived seriousness, perceptions about the likelihood of pain persistence and whether they would result in being fearful of movement. Our secondary aim was to describe the association between self-reported understanding of the terms and back beliefs, and history and intensity of LBP. We also elicited views about access to imaging reports and whether they should be understandable to patients and include context.

METHODS

Design

This study was a cross-sectional online survey of a random sample of the general public in five English-speaking countries: Australia, New Zealand, the United States, United Kingdom, Canada. It was conducted and reported in accordance with the Checklist for Reporting Results of Internet E-Surveys (CHERRIES) guideline (18).

Participants and recruitment

Participants were recruited in April 2019 via an online survey sampling company (Dynata). Dynata is a data company that maintains a research and marketing database. It recruits participants via email based on the requirements of the research involved. Upon survey completion, Dynata provides participant incentives that allow redemption of items such as gift cards and contributions to charity. English-speaking adults aged 18 and over with or without a

For peer review only

current or past history of back pain were recruited via email with quotas established for country, age and gender to ensure a representative sample.

The survey was conducted in Qualtrics and no identifying information was provided to the research team.

Survey instrument

Pilot testing conducted in a convenience sample of ten members of the general public resulted in minor changes. The survey included nine demographic questions (age in years, gender (female/male/other/prefer not to say), education level (finished high school/technical/trade certificate or diploma, university (Bachelor), post-graduate university qualifications), employment status (full time/part time/unemployed/student/home duties/retired (not due to ill health)/retired (due to ill health)/other), health care provider for people with LBP (yes/no), LBP history (current, past, never) and for those with a history of LBP we asked about average pain intensity (measured on a 0 to 100 point VAS where higher scores indicate greater pain), LBP imaging (Yes/No/Don't Know) and treatment (surgery and injections).

Four statements were presented for each of 14 radiology terms (annular fissure, disc bulge, disc degeneration, disc extrusion, disc height loss, disc protrusion, disc signal loss, facet joint degeneration, high intensity zone, mild canal stenosis, modic changes, nerve root contact, spondylolisthesis and spondylosis). These terms are commonly found in lumbar imaging reports of people with LBP but are also common findings in asymptomatic populations and considered benign or of unclear clinical significance.(6, 19, 20) For each question, order of presentation of the terms was randomised to reduce order effect bias.

For peer review only - http://bmjopen.bmj.com/site/about/guidelines.xhtml

For each item, we elicited the participant's level of agreement on a 5-point Likert scale (1=Completely disagree, 2=Somewhat disagree, 3=Neither agree nor disagree, 4=Somewhat agree, 5=Completely agree) to the following four statements:

1. I would need to look this term up to know what it means (Understanding)
2. I would be worried there is a serious problem with my back (Seriousness)
3. I would be worried my pain isn't going to get any better (Persistence)
4. I would be afraid to move my back in case I did more damage (Fear of movement)

Items 2 and 3 were derived from questionnaires for musculoskeletal conditions or LBP that assess perceived seriousness or likely persistence of pain.(21-23) For example the third item is similar to items in the STarT Back Tool ('I feel my back pain is terrible and it's never going to get any better'),(24) Pain Catastrophizing Scale ('It's terrible and I think it's never going to get any better')(25) and the Back-PAQ ('There is a high chance that an episode of back pain will not resolve').(22)

We also elicited opinions (yes/no/don't know) about whether people should have access to their imaging reports; whether imaging reports should be written so they can be understood by lay people; and whether including epidemiological information about common findings in people without back pain would be helpful in interpreting results.

The Back Beliefs Questionnaire (BBQ) is a 14 item tool that measures beliefs about back pain.(26) Responses to each item are measured on a 5-point Likert agreement scale (completely disagree to completely agree). The BBQ is scored from nine of the 14 items with higher scores indicating more positive beliefs (score range 9 to 45).

For peer review only

Sample size and analysis

A minimum sample size of 590 was required to detect 70% ± 10% prevalence of not understanding a term. Assuming 10% drop out or non-completion of surveys, our target was 650 participants. This sample size also provided enough power to investigate associations between variables.

Raw data files were reviewed for evidence of duplicate answers including duplicate internet protocol (IP) addresses prior to inclusion in the analysis. Descriptive statistics were used to summarise the participant characteristics, responses to statements about radiological terms, back beliefs and opinions regarding imaging reports. Responses of either completely disagree or somewhat disagree with the statement 'I would need to look this up to know what it means' were considered to indicate self-reported understanding of a term.

Responses for each item were dichotomised with ‘completely disagree’ and ‘somewhat disagree’ coded as zero and ‘neither agree nor disagree’/’somewhat agree’/’completely agree’ coded as one. These dichotomised scores were summed into total response numbers for each participant for the 14 radiology terms ranging from 0 to 14. For the item about understanding, a score of 0 indicates that respondents reported that they would not need to look up any of the 14 terms (high level of understanding), while a score of 14 indicates they would need to look up all terms (low understanding). For items regarding worry about seriousness, persistence of pain and fear of movement, a score of 0 indicates that none of the terms elicited worry about seriousness, persistence of pain and/or movement, while a score of 14 indicates that all terms elicited concerns about seriousness, persistence of pain and/or movement. Following distribution assessment of scores, they were reversed.

For peer review only - http://bmjopen.bmj.com/site/about/guidelines.xhtml

After assessment of best fit via the 'nbvargr' Stata command, multivariate negative binomial regression was used to investigate the association between self-reported understanding of terms and perceived seriousness, risk of persistence and fear of movement, and their association with participant demographics, history of LBP and BBQ score. Spearman's rank correlation was used to investigate the correlation between responses to the four statements regarding the terms.

Ethics

This project was approved by the Monash University Human Research Ethics Committee (ID 13896). Survey completion was considered consent to participate and participants were able to review their answers prior to submission.

Patient and public involvement

Consumer feedback about questionnaire burden and time requirements was incorporated into survey design. Survey participants were advised how to access results of the study in the accompanying explanatory statement.

RESULTS

Of 774 responses, we excluded 97 (12.5%) due to incomplete data (n=35, 4.5%), invalid BBQ responses (n=59, 7.6%) and age given as under 18 years (n=3, 0.4%). No duplicate responses were identified.

Responses from 677 participants (52% female) were analysed (Table 1). There were no significant between-country differences regarding demographic characteristics. 577 participants (85%) reported past or current LBP, and 279 (41%) reported having LBP at the

For peer review only

Table 1: Participant demographics and back pain history and beliefs by country and overall

		USA (n=139)	Australia (n=138)	NZ (n=135)	UK (n=132)	Canada (n=133)	TOTAL (n=677)
		Mean (SD)	*Mean (SD)*	*Mean (SD)*	*Mean (SD)*	*Mean (SD)*	*Mean (SD)*
Age, years		45.4 (16.0)	45.0 (17.0)	45.9 (17.6)	47.6 (17.0)	45.4 (15.5)	45.8 (16.4)
Back pain intensity most recent episode* (Scale 0-100, higher score indicates greater pain)	Current LBP	49.7 (22.4)	58.2 (20.2)	55.0 (21.7)	58.1 (22.0)	51.9 (20.2)	54.7 (21.3)
	Previous LBP	40.0 (19.7)	48.4 (22.6)	44.2 (21.3)	45.4 (24.4)	44.0 (22.6)	44.2 (22.1)
BBQ score (Scale 9-45, higher score indicates better back beliefs)		25.7 (6.4)	24.3 (6.5)	25.8 (6.6)	24.6 (6.3)	24.4 (6.1)	25.0 (6.4)
		N (%)	*N (%)*	*N (%)*	*N (%)*	*N (%)*	*N (%)*
Female		75 (54)	73 (53)	70 (52)	67 (51)	69 (52)	354 (52)
University education		69 (50)	51 (37)	63 (47)	59 (45)	57 (43)	299 (44)
Employed full or part time		71 (51)	80 (58)	77 (57)	73 (55)	80 (60)	381 (56)
Worked as healthcare professional		10 (7)	4 (3)	4 (3)	5 (4)	4 (3)	27 (4)
Ever had low back pain		116 (83)	119 (86)	114 (84)	113 (86)	115 (86)	577 (85)
Current low back pain		54 (38)	68 (49)	45 (33)	52 (39)	60 (45)	279 (41)
Had lumbar spine imaging*		60 (52)	60 (50)	40 (35)	38 (34)	53 (46)	251 (44)
Had lumbar spine injection*		17 (15)	18 (15)	16 (14)	21 (19)	9 (8)	81 (14)
Had lumbar spine surgery*		6 (5)	4 (3)	7 (6)	6 (5)	4 (4)	27 (5)

*Only for participants who responded 'yes' to current or previous history of low back pain

Abbreviations: USA – United States of America, NZ – New Zealand, UK – United Kingdom, SD – Standard Deviation, BBQ – Back beliefs questionnaire, LBP – Low back pain

For peer review only - http://bmjopen.bmj.com/site/about/guidelines.xhtml

time of survey completion. The mean (SD) BBQ score for the whole cohort was 25.0 (6.4). Pain intensity was higher in those who reported current compared with past LBP (mean (SD): 54.7 (21.3), n=279, versus 44.2 (22.1), n=298, respectively, p<0.001). Of those with a history of LBP, 251 (44%) reported receipt of lumbar spine imaging, 81 (14%) reported receipt of spine injection/s and 27 (5%) reported undergoing spine surgery.

Participant views regarding terms

Most participants (n=598, 88%) somewhat or completely agreed they would need to look up at least one of the terms to know what it means, while only 3% (n=17) somewhat or completely disagreed with this statement for all terms (Figure 1). Overall, 41 (6%) participants provided neutral answers for all questions (i.e., neither agree nor disagree).

The proportion who reported understanding the meaning of terms ranged from 35% (n=235) for the term 'disc degeneration' to 10.5% (n=71) for 'modic changes' (Figure 1a). The same proportion (88%) somewhat or completely agreed they would be worried about a serious problem with their back for at least one term and only 3% (n=17) disagreed with this statement for all terms (Figure 1b). Three terms, 'disc extrusion', 'disc degeneration' and 'facet joint degeneration', elucidated the most worry about seriousness (71% of participants). Worry regarding seriousness varied only a small amount between terms, with the number of participants disagreeing with the statement varying between only 7% and 11% for all terms.

For all terms, over half the participants were worried that back pain would persist if the imaging finding was present (Figure 1c). The least number of participants (51%) reported worry regarding persistence for the term 'modic changes', while 'disc degeneration' was the term which caused worry in the greatest percent of participants (71%). In total 84% of

For peer review only - http://bmjopen.bmj.com/site/about/guidelines.xhtml

participants agreed they would be worried regarding persistence for at least one term, and 4% of participants disagreed with the statement for all terms.

For many imaging findings, participants also frequently reported they would be afraid to move their back. 74% agreed with this statement for at least one term and only 8% disagreed with this statement for all terms (Figure 1d). The greatest number of participants agreed with this statement for the term ‘disc bulge’ (57%), and 56% of participants also agreed with this statement for the terms ‘disc degeneration’, ‘disc protrusion’, ‘nerve root contact’ and ‘disc extrusion’. ‘Modic changes’ and ‘mild canal stenosis’ were the terms associated with least worry regarding movement (42% agreed with the statement).

Associations between self-reported understanding of terms and perceived seriousness, risk of pain persistence and fear of movement

There was moderate correlation between understanding of terms and worry about seriousness, pain persistence and fear of movement (Spearman’s rho 0.48, $p<0.001$, 0.39, $p<0.001$ and 0.38, $p<0.001$ respectively) (Table 2). Strong associations were present between the responses regarding worry about seriousness and pain persistence (Spearman’s rho 0.63, $p<0.001$) and pain persistence and fear of movement (0.51, $p<0.001$).

Table 2: Association between the self-reported understanding of the 14 terms overall and perceived seriousness, pain persistence and fear of movement, Spearman’s rho correlation*

	Understanding	Seriousness	Pain persistence	Fear of movement
**Understanding**	1			
**Seriousness**	0.48	1		
**Persistence**	0.39	0.63	1	
**Fear of movement**	0.38	0.42	0.51	1

**All p values <0.001.*

Values between 0.10 and 0.29 represent a weak association, 0.30-0.49 moderate and 0.5 and above a strong association.

For peer review only - http://bmjopen.bmj.com/site/about/guidelines.xhtml

Table 3: Association between self-reported understanding of the 14 terms, perceived seriousness, pain persistence and fear of movement and demographic details, history of back pain and back beliefs

	**Understanding**	**Seriousness**	**Pain persistence**	**Fear of movement**
	Regression coefficient (95% Confidence Interval)			
Education (REF - High school)				
Trade/Diploma	**0.32 (0.07, 0.57)***	-0.27 (-0.57, 0.03)	**-0.73 (-1.02 to -0.43)*****	**-0.37 (-0.63, -0.11)****
Bachelor	**0.34 (0.12, 0.57)****	**- 0.35 (-0.62, -0.09)****	**-0.38 (-0.65 to -0.11)****	**-0.44 (-0.68 to -0.20)*****
Postgraduate qualifications	**0.51 (0.23, 0.80)*****	**-0.69 (-1.01, -0.36)*****	**-0.98 (-1.31, -0.66)*****	**-0.61 (-0.91, -0.32)*****
LBP History (REF - Never)				
Previous LBP	0.20 (-0.08, 0.48)	0.27 (-0.04, 0.57)	0.01 (-0.30 to 0.32)	**-0.32 (-0.61, -0.02)***
Current LBP	**0.42 (0.14, 0.70)****	0.10 (-0.22, 0.42)	-0.13 (-0.45, 0.19)	**-0.61 (-0.91, -0.31)*****
BBQ score	**0.02 (0.004, 0.03)***	**-0.03 (-0.05 to -0.02)*****	**-0.06 (-0.08 to -0.04)*****	**-0.07 (-0.08, -0.05)*****
Age, years	0.002 (-0.004, 0.01)	**0.02 (0.01, 0.03)*****	**0.02 (0.02, 0.03)*****	**-0.007 (-0.01, -0.001)***
Gender (REF - Female)				
Male	0.06 (-0.12, 0.24)	**-0.31 (-0.52, -0.09)****	-0.19 (-0.40, 0.03)	**-0.21 (-0.40, -0.02)***

Note: Multivariate negative binomial regression results – bold indicates statistically significant, * $p < 0.05$, **$p < 0.01$, ***$p < 0.001$. Results rounded to two decimal points or first meaningful decimal point. REF = Reference category, LBP = Low Back Pain, BBQ = Back Beliefs Questionnaire

Continuous variables (BBQ score and age): For understanding, a positive regression coefficient indicates greater self-reported understanding is associated with better back beliefs (higher BBQ score) and increased age. For seriousness, pain persistence and fear of movement, a positive regression coefficient indicates greater worry/concern is associated with poorer back beliefs (lower BBQ score) and increased age.

Categorical variables: For understanding, a positive regression coefficient indicates increased self-reported understanding compared with the reference category. For seriousness, pain persistence and fear of movement, a positive regression coefficient indicates greater worry/concern compared with the reference variable.

For peer review only - http://bmjopen.bmj.com/site/about/guidelines.xhtml

Associations with demographic features, history of back pain and back beliefs

Results of the regression analysis are presented in Table 3. Higher education level was associated with greater self-reported understanding of terms, and less concern about seriousness, pain persistence and being fearful of movement. Participants with current LBP had better understanding and less fear of movement compared with those without a history of LBP. More positive back beliefs (higher BBQ score) were associated with greater self-reported understanding of terms and less worry regarding seriousness, persistence and movement. Older age was associated with greater worry about seriousness and pain persistence but less fear of movement. Male gender was associated with less worry about seriousness and movement compared with female gender.

Participants views about imaging reports

The majority of participants (n=605, 89%) agreed that people with LBP should have access to their radiology report, that it should be written in a way to facilitate lay understanding (n=586, 87%) and it would be useful to include information about the prevalence of common imaging findings in people without back pain (n=587, 87%).

Discussion

Our study found evidence of poor self-reported understanding of terms commonly used to describe usually benign findings reported in lumbar spine imaging reports. Most participants had concerns about their seriousness and associated the terms with persistence of pain and/or need to avoid (or be fearful of) movement. Higher education, more positive back beliefs and current LBP were all associated with greater self-reported understanding of the terms, while people with higher education and more positive back beliefs were also less concerned about seriousness, pain persistence and being fearful of movement.

For peer review only - http://bmjopen.bmj.com/site/about/guidelines.xhtml

Our findings, consistent with other literature that has found that language matters, (15, 16) provide support for changing how these findings are reported. Recent online scenario-based randomised experiments that provided imaging reports for 'virtual patients' with LBP found that altering the language and/or providing epidemiologic information improved perceptions regarding LBP compared with provision of a standard report.(27, 28) This now requires confirmation in randomised trials of real patients.

The radiology report may play a role in mediating the relationship between imaging and overtreatment in LBP, (7, 29) and providing contextual information has been reported to reduce unnecessary repeat imaging, opioid prescription and specialist referral.(30, 31) Trials that have assessed the provision of epidemiologic data or additional educational messaging,(32-36) including for LBP,(20, 36, 37) have been designed to improve referrer knowledge and behaviour, but none have assessed patient knowledge or other outcomes. Our study provides evidence that commonly identified and reported terms in radiology reports are also associated with misplaced patient concerns about their implications, which may also influence their healthcare decision-making.

Those with current LBP had poorer back beliefs compared to those without a history of LBP or past LBP. This is consistent with previous studies that have found greater pessimism regarding recovery among those with current symptoms.(26, 38-41) This may also explain their increased expectation and wish for further tests and treatment.(42)

For peer review only - http://bmjopen.bmj.com/site/about/guidelines.xhtml

Strengths and limitations

To our knowledge this is the first study to measure societal self-reported understanding of commonly used terms in lumbar spine imaging reports and their relationship to other factors such as perceived seriousness, persistence of pain and fear of movement. Our large representative sample from five countries indicates our results are likely to be generalisable as we used quotas to reflect the make-up of the respective populations. Additionally, the proportion of participants with a history of LBP is consistent with other population studies,(43) with population BBQ scores similar to other cohorts.(38, 40, 41, 44, 45) Our study was appropriately powered and randomisation of the terms for each participant minimised any order effect bias. Where possible we adapted our survey items from other tools with established validity and reliability and the survey was piloted prior to use.

Survey measurement of public understanding of medical terms is challenging. Asking respondents whether they would need to look the term up to know what it means could be considered an even more indirect measure of self-reported understanding. As well, self-reported understanding of a medical term may not be a true reflection of participants' true understanding of terms and our study may have underestimated true understanding. We used a survey sampling company, which may have introduced participation bias as participants receive rewards albeit very small ones. They also represent people who are online and familiar with computers and online questionnaires.

All of the imaging findings we assessed frequently exist in the asymptomatic population and therefore may or may not have relevance in an individual case. However, some findings such as disc bulge, disc extrusion, Modic changes, disc protrusion and disc degeneration, occur

For peer review only - http://bmjopen.bmj.com/site/about/guidelines.xhtml

more commonly in symptomatic populations.(19) Participants' past experiences with LBP and/or imaging of the lumbar spine and these terms may have influenced their responses.

Implications for practice

All terms we investigated elicited concerns that were likely unwarranted. Combined with our finding that most participants would like access to their reports, more consideration is needed about how to report findings in lumbar imaging reports in people with LBP. Our finding that respondents wanted reports to be understandable to the lay person is also consistent with the results of other studies.(46, 47)

Although clinicians may have reservations about allowing patients direct access to their medical information,(48) there is already increased availability and use of electronic medical records (49) and in many health systems this is standard practice.(50) There is now both imperative and empirical data indicating the importance of accurately and clearly portraying the significance of imaging findings in terms that are understandable to both clinicians and patients.

CONCLUSION

Common and usually non-serious terms in lumbar spine imaging reports are poorly understood by the general population and may contribute to the burden of LBP. Incorporating clear explanations about the implications of these findings may reduce unwarranted anxiety and reduce low-value care.

For peer review only

Contributorship statement

All authors contributed to the conception and/or design of this study. CF, DO, RB, HL and CM made substantial contributions to the analysis and interpretation of the data. All authors contributed to drafts and critical appraisal of the manuscript, and approved the final version. All authors agree to be accountable for all aspects of this study.

We acknowledge and thank Alex Gorelik for her assistance with data analysis for this study.

Competing interests statement

No competing interests relating to this publication have been declared. CM and RB have received grants from numerous government and not-for-profit agencies; their expenses have been covered by professional associations hosting conferences where they were speakers, and they were both investigators for a trial that received heat wraps from Flexeze at no cost.

Funding statement

DO is supported by an Australian National Health and Medical Research Council (NHMRC) Translating Research into Practice Fellowship (APP1168749). HL is funded by an NHMRC (grant no. APP1126767); receives project funding from the Berkeley Initiative for Transparency in the Social Sciences, a program of the Center for Effective Global Action (CEGA), with support from the Laura and John Arnold Foundation. He is also supported by funding from the National Institute for Health Research (NIHR) Collaboration for Leadership in Applied Health Research and Care Oxford at Oxford Health NHS Foundation Trust. JGJ is supported by funding from the US National Institute of Health/NIAMS (P30AR072572). CM is supported by an NHMRC Principal Research Fellowship (APP1103022). RB is supported by an NHMRC Senior Principal Research Fellowship (APP1082138).

For peer review only - http://bmjopen.bmj.com/site/about/guidelines.xhtml

For peer review only

Data sharing statement

The study protocol and deidentified participant data is available upon reasonable request to the corresponding author (caitlin.farmer1@monash.edu).

Figure legend/caption

Figure 1: Responses to questions regarding level of understanding, seriousness, persistence of pain and fear of movement for the 14 radiology terms

Note: For each term, we asked four questions with a common opening ('If this term was in my report I would...') to evaluate a) understanding ('....need to look this term up to know what it means'); b) worry about seriousness ('...be worried there is a serious problem with my back'); c) worry about persistence ('...be worried my pain would persist') and d) fear of movement ('...be afraid to move in case I did more damage').

For peer review only

References

1. Hartvigsen J, Hancock MJ, Kongsted A, et al. What low back pain is and why we need to pay attention. *The Lancet*. 2018. doi:10.1016/s0140-6736(18)30480-x

2. Maher C, Underwood M, Buchbinder R. Non-specific low back pain. *The Lancet*. 2017;389(10070):736-47. doi:10.1016/s0140-6736(16)30970-9

3. National Institute for Health and Care Excellence: Clinical Guidelines. Low Back Pain and Sciatica in Over 16s: Assessment and Management. London: National Institute for Health and Care Excellence (UK); 2016.

4. Chou R, Qaseem A, Snow V, et al. Diagnosis and treatment of low back pain: A joint clinical practice guideline from the american college of physicians and the american pain society. *Annals of Internal Medicine*. 2007;147(7):478-91. doi:10.7326/0003-4819-147-7-200710020-00006

5. Oliveira CB, Maher CG, Pinto RZ, et al. Clinical practice guidelines for the management of non-specific low back pain in primary care: an updated overview. *European Spine Journal*. 2018;27(11):2791-803. doi:10.1007/s00586-018-5673-2

6. Brinjikji W, Luetmer PH, Comstock B, et al. Systematic literature review of imaging features of spinal degeneration in asymptomatic populations. *AJNR Am J Neuroradiol*. 2015;36(4):811-6. doi:10.3174/ajnr.A4173

7. Webster BS, Choi Y, Bauer AZ, et al. The cascade of medical services and associated longitudinal costs due to nonadherent magnetic resonance imaging for low back pain. *Spine (Phila Pa 1976)*. 2014;39(17):1433-40. doi:10.1097/BRS.0000000000000408

8. Webster BS, Bauer AZ, Choi Y, et al. Iatrogenic consequences of early magnetic resonance imaging in acute, work-related, disabling low back pain. *Spine (Phila Pa 1976)*. 2013;38(22):1939-46. doi:10.1097/BRS.0b013e3182a42eb6

For peer review only - http://bmjopen.bmj.com/site/about/guidelines.xhtml

9. McCaffery K, Nickel B, Moynihan R, et al. How different terminology for ductal carcinoma in situ impacts women's concern and treatment preferences: a randomised comparison within a national community survey. *BMJ Open*. 2015;5(11):e008094. doi:10.1136/bmjopen-2015-008094

10. Copp T, McCaffery K, Azizi L, et al. Influence of the disease label 'polycystic ovary syndrome' on intention to have an ultrasound and psychosocial outcomes: a randomised online study in young women. *Hum Reprod*. 2017;32(4):876-84. doi:10.1093/humrep/dex029

11. Scherer LD, Finan C, Simancek D, et al. Effect of "Pink Eye" Label on Parents' Intent to Use Antibiotics and Perceived Contagiousness. *Clin Pediatr (Phila)*. 2016;55(6):543-8. doi:10.1177/0009922815601983

12. Scherer LD, Zikmund-Fisher BJ, Fagerlin A, et al. Influence of "GERD" label on parents' decision to medicate infants. *Pediatrics*. 2013;131(5):839-45. doi:10.1542/peds.2012-3070

13. Scherer AM, Knaus M, Zikmund-Fisher BJ, et al. Effects of Influenza Strain Label on Worry and Behavioral Intentions. *Emerg Infect Dis*. 2017;23(8):1425-6. doi:10.3201/eid2308.170364

14. Azam N, Harrison M. Patients' perspectives on injuries. *Emerg Med J*. 2011;28(7):601-3. doi:10.1136/emj.2009.082032

15. Nickel B, Barratt A, Copp T, et al. Words do matter: a systematic review on how different terminology for the same condition influences management preferences. *BMJ Open*. 2017;7(7):e014129. doi:10.1136/bmjopen-2016-014129

16. Rosenkrantz AB. Differences in Perceptions Among Radiologists, Referring Physicians, and Patients Regarding Language for Incidental Findings Reporting. *AJR Am J Roentgenol*. 2017;208(1):140-3. doi:10.2214/AJR.16.16633

For peer review only

For peer review only - http://bmjopen.bmj.com/site/about/guidelines.xhtml

17. Bossen JK, Hageman MG, King JD, et al. Does rewording MRI reports improve patient understanding and emotional response to a clinical report? *Clin Orthop Relat Res*. 2013;471(11):3637-44. doi:10.1007/s11999-013-3100-x

18. Eysenbach G. Improving the quality of Web surveys: the Checklist for Reporting Results of Internet E-Surveys (CHERRIES). *Journal of medical Internet research*. 2004;6(3):e34-e. doi:10.2196/jmir.6.3.e34

19. Brinjikji W, Diehn FE, Jarvik JG, et al. MRI Findings of Disc Degeneration are More Prevalent in Adults with Low Back Pain than in Asymptomatic Controls: A Systematic Review and Meta-Analysis. *AJNR Am J Neuroradiol*. 2015;36(12):2394-9. doi:10.3174/ajnr.A4498

20. Jarvik JG, Comstock BA, James KT, et al. Lumbar Imaging With Reporting Of Epidemiology (LIRE)--Protocol for a pragmatic cluster randomized trial. *Contemp Clin Trials*. 2015;45(Pt B):157-63. doi:10.1016/j.cct.2015.10.003

21. Linton SJ, Boersma K. Early Identification of Patients at Risk of Developing a Persistent Back Problem: The Predictive Validity of The Örebro Musculoskeletal Pain Questionnaire. *The Clinical Journal of Pain*. 2003;19(2):80-6.

22. Darlow B, Perry M, Mathieson F, et al. The development and exploratory analysis of the Back Pain Attitudes Questionnaire (Back-PAQ). *BMJ Open*. 2014;4(5).

23. Robinson HS, Dagfinrud H. Reliability and screening ability of the StarT Back screening tool in patients with low back pain in physiotherapy practice, a cohort study. *BMC Musculoskeletal Disorders*. 2017;18:232. doi:10.1186/s12891-017-1553-x

24. Hill JC, Whitehurst DGT, Lewis M, et al. Comparison of stratified primary care management for low back pain with current best practice (STarT Back): a randomised controlled trial. *The Lancet*. 2011;378(9802):1560-71. doi:10.1016/s0140-6736(11)60937-9

For peer review only

25. Sullivan MJ, Bishop SR. The Pain Catastrophizing Scale: Development and Validation.

26. Bostick GP, Schopflocher D, Gross DP. Validity evidence for the back beliefs questionnaire in the general population. *Eur J Pain*. 2013;17(7):1074-81. doi:10.1002/j.1532-2149.2012.00275.x

27. Karran EL, Medalian Y, Hillier SL, et al. The impact of choosing words carefully: an online investigation into imaging reporting strategies and best practice care for low back pain. *PeerJ*. 2017;5:e4151. doi:10.7717/peerj.4151

28. Medalian Y, Moseley GL, Karran EL. An online investigation into the impact of adding epidemiological information to imaging reports for low back pain. *Scandinavian Journal of Pain*. 2019;19(3):629. doi:https://doi.org/10.1515/sjpain-2019-0023

29. Lemmers GPG, van Lankveld W, Westert GP, et al. Imaging versus no imaging for low back pain: a systematic review, measuring costs, healthcare utilization and absence from work. *Eur Spine J*. 2019. doi:10.1007/s00586-019-05918-1

30. Fried JG, Andrew AS, Ring NY, et al. Changes in Primary Care Health Care Utilization after Inclusion of Epidemiologic Data in Lumbar Spine MR Imaging Reports for Uncomplicated Low Back Pain. *Radiology*. 2018;287(2):563-9. doi:10.1148/radiol.2017170722

31. McCullough BJ, Johnson GR, Martin BI, et al. Lumbar MR imaging and reporting epidemiology: do epidemiologic data in reports affect clinical management? *Radiology*. 2012;262(3):941-6. doi:10.1148/radiol.11110618

32. Heidenreich PA, Gholami P, Sahay A, et al. Clinical reminders attached to echocardiography reports of patients with reduced left ventricular ejection fraction increase use of beta-blockers: a randomized trial. *Circulation*. 2007;115(22):2829-34. doi:10.1161/CIRCULATIONAHA.106.684753

For peer review only - http://bmjopen.bmj.com/site/about/guidelines.xhtml

33. Heidenreich PA, Chacko M, Goldstein MK, et al. ACE inhibitor reminders attached to echocardiography reports of patients with reduced left ventricular ejection fraction. *Am J Med*. 2005;118(9):1034-7. doi:10.1016/j.amjmed.2004.12.028

34. Stock JL, Wand CE, Coderre JA, et al. Clinical Reporting to Primary Care Physicians Leads to Increased Use and Understanding of Bone Densitometry and Affects the Management of Osteoporosis. *Annals of Internal Medicine*. 1998;128(12_Part_1). doi:10.7326/0003-4819-128-12_Part_1-199806150-00006

35. Ramsay CR, Eccles M, Grimshaw JM, et al. Assessing the long-term effect of educational reminder messages on primary care radiology referrals. *Clinical Radiology*. 2003;58(4):319-21.

36. Eccles M, Steen N, Grimshaw J, et al. Effect of audit and feedback, and reminder messages on primary-care radiology referrals: a randomised trial. *Lancet*. 2001;357(9266):1406-9.

37. Jarvik JG, Meier EN, James KT, et al. The Effect of Including Benchmark Prevalence Data of Common Imaging Findings in Spine Image Reports on Health Care Utilization Among Adults Undergoing Spine Imaging: A Stepped-Wedge Randomized Clinical Trial. *JAMA Network Open*. 2020;3(9):e2015713-e. doi:10.1001/jamanetworkopen.2020.15713

38. Gross DP, Ferrari R, Russell AS, et al. A population-based survey of back pain beliefs in Canada. *Spine (Phila Pa 1976)*. 2006;31(18):2142-5. doi:10.1097/01.brs.0000231771.14965.e4

39. Darlow B, Perry M, Stanley J, et al. Cross-sectional survey of attitudes and beliefs about back pain in New Zealand. *BMJ Open*. 2014;4(5):e004725. doi:10.1136/bmjopen-2013-004725

For peer review only

40. Buchbinder R, Jolley D. Effects of a media campaign on back beliefs is sustained 3 years after its cessation. *Spine (Phila Pa 1976)*. 2005;30(11):1323-30. doi:10.1097/01.brs.0000164121.77862.4b

41. Buchbinder R, Jolley D, Wyatt M. Population based intervention to change back pain beliefs and disability: three part evaluation. *BMJ*. 2001;322(7301):1516-20. doi:10.1136/bmj.322.7301.1516

42. Jenkins HJ, Hancock MJ, Maher CG, et al. Understanding patient beliefs regarding the use of imaging in the management of low back pain. *Eur J Pain*. 2016;20(4):573-80. doi:10.1002/ejp.764

43. Hoy D, Brooks P, Blyth F, et al. The Epidemiology of low back pain. *Best Pract Res Clin Rheumatol*. 2010;24(6):769-81. doi:10.1016/j.berh.2010.10.002

44. Buchbinder R, Jolley D. Population based intervention to change back pain beliefs: three year follow up population survey. *BMJ*. 2004;328(7435):321. doi:10.1136/bmj.328.7435.321

45. Gross DP, Russell AS, Ferrari R, et al. Evaluation of a Canadian Back Pain Mass Media Campaign. *Spine*. 2010;35(8):906-13. doi:10.1097/BRS.0b013e3181c91140

46. Cabarrus M, Naeger DM, Rybkin A, et al. Patients Prefer Results From the Ordering Provider and Access to Their Radiology Reports. *J Am Coll Radiol*. 2015;12(6):556-62. doi:10.1016/j.jacr.2014.12.009

47. Johnson AJ, Easterling D, Williams LS, et al. Insight from patients for radiologists: improving our reporting systems. *J Am Coll Radiol*. 2009;6(11):786-94. doi:10.1016/j.jacr.2009.07.010

48. Johnson AJ, Frankel RM, Williams LS, et al. Patient Access to Radiology Reports: What Do Physicians Think? *J*. 2010;7(4):281-9. doi:http://dx.doi.org/10.1016/j.jacr.2009.10.011

For peer review only

For peer review only - http://bmjopen.bmj.com/site/about/guidelines.xhtml

49. Henshaw D, Okawa G, Ching K, et al. Access to radiology reports via an online patient portal: Experiences of referring physicians and patients. *J*. 2015;12(6):582-6.e1. doi:http://dx.doi.org/10.1016/j.jacr.2015.01.015

50. Zanaboni P, Ngangue P, Mbemba GIC, et al. Methods to Evaluate the Effects of Internet-Based Digital Health Interventions for Citizens: Systematic Review of Reviews. *J Med Internet Res*. 2018;20(6):e10202. doi:10.2196/10202

For peer review only

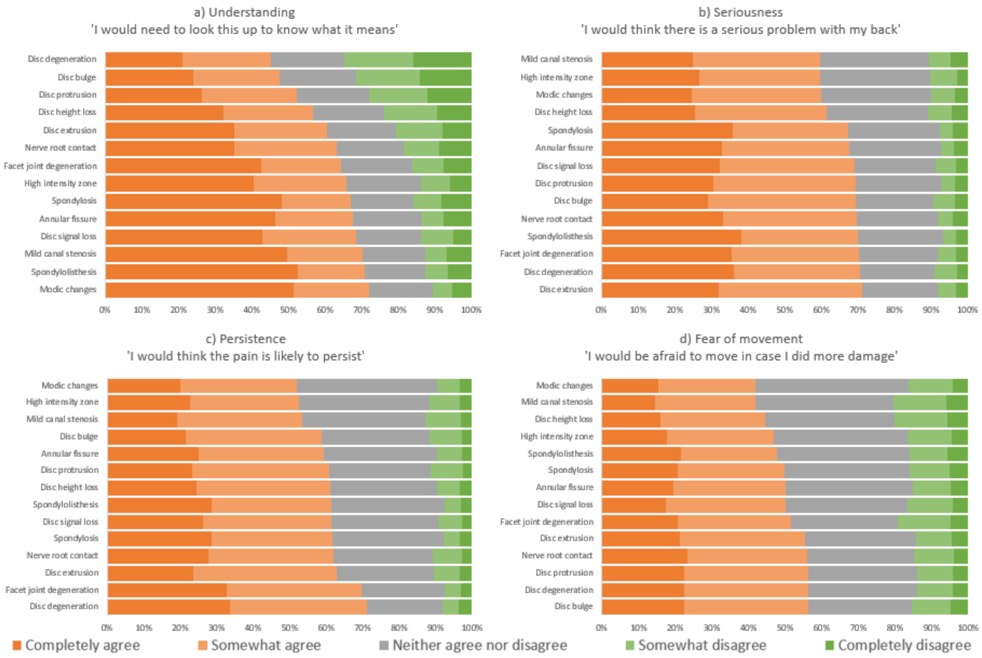

Responses to questions regarding level of understanding, seriousness, persistence of pain and fear of movement for the 14 radiology terms

Note: For each term, we asked four questions with a common opening ('If this term was in my report I would...') to evaluate a) understanding ('....need to look this term up to know what it means'); b) worry about seriousness ('...be worried there is a serious problem with my back'); c) worry about persistence ('...be worried my pain would persist') and d) fear of movement ('...be afraid to move in case I did more damage').

Cherries checklist – Consumer understanding of terms used in imaging reports requested for low back pain

	Category	Checklist item	Page(s)
1	Design	**Describe survey design**<br>Describe target population, sample frame. Is the sample a convenience sample? (In 'open' surveys this is most likely).	5-9
2	IRB (Institutional Review Board) approval and informed consent process	**IRB approval**<br>Mention whether the study has been approved by an IRB.	9
3		**Informed consent**<br>Describe the informed consent process. Where were the participants told the length of time of the survey, which data were stored and where and for how long, who the investigator was, and the purpose of the study?	9
4		**Data protection**<br>If any personal information was collected or stored, describe what mechanisms were used to protect unauthorised access.	N/A
5	Development and pre-testing	**Development and testing**<br>Stated how the survey was developed, including whether the usability and technical functionality of the electronic questionnaire had been tested before fielding the questionnaire	6
6	Recruitment process and description of the sample having access to the questionnaire	**Open survey vs closed survey**<br>An 'open survey' is a survey open for each visitor of a site, while a closed survey is only open to a sample which the investigator knows (password-protected survey).	5-6
7		**Contact mode**<br>Indicate whether or not the initial contact with the potential participants was made on the internet (Investigators may also send out questionnaires by mail and allow for Web-based data entry).	5-6
8		**Advertising the survey**<br>How/where was the survey announced or advertised? Some examples are offline media (newspapers), or online (mailing lists – if yes, which ones?) or banner ads (where were these banner ads posted and what did they look like?). It is important to know the wording of the announcement as it will heavily influence who chooses to participate. Ideally the survey announcement should be published as an appendix.	5-6
9	Survey administration	**Web/email**<br>State the type of e-survey (eg one posted on a website, or one sent out through email). If it is an email survey, were the responses entered manually into a database, or was there an automatic method for capturing responses?	5
10		**Context**<br>Describe the website (for mailing list/newsgroup) in which the survey was posted. What is the website about, who is visiting it, what are visitors normally looking for? Discuss to what degree the content of the website could pre-select	N/A

For peer review only

		the sample or influence the result. For example, a survey about vaccination on an anti-immunisation website will have different results from a web survey conducted on a N/A government website.	
11		**Mandatory/voluntary**<br>Was it a mandatory survey to be filled in by every visitor who wanted to enter the website, or was it a voluntary survey?	N/A
12		**Incentives**<br>Were any incentives offered (eg monetary, prizes, or non-monetary incentives such as an offer to provide the survey results)?	5-6
13		**Time/Date**<br>In what timeframe were the data collected?	5
14		**Randomisation of items or questionnaires**<br>To prevent biases items can be randomised or alternated	6
15		**Adaptive questioning**<br>Use adaptive questioning (certain items, or only conditionally displayed based on responses to other items) to reduce number and complexity of the questions	5-6
16		**Number of items**<br>What was the number of questionnaire items per page? The number of items is an important factor for the completion rate.	6-7
17		**Number of screens (pages)**<br>Over how many pages was the questionnaire distributed? The number of items is an important factor for the completion rate.	N/A
18		**Completeness check**<br>It is technically possible to do consistency or completeness checks before the questionnaire is submitted. Was this done, and if 'yes' how (usually JAVAscript)? An alternative is to check for completeness after the questionnaire has been submitted (and highlight mandatory items). If this has been done, it should be reported. All items should provide a non-response option such as 'not applicable' or 'rather not say' and selection of one response option should be enforced.	9
19		**Review step**<br>State whether respondents were able to review and change their answers (eg through a Back button or a Review step which displays a summary of the responses and asks the respondents if they are correct).	9
20	Response rates	**Unique site visitor**<br>If you provide view rates or participation rates, you need to define how you determined a unique visitor. There are different techniques available, based on IP addresses or cookies or both.	N/A

For peer review only

21		**View rate**<br>**(ratio of unique survey visitors/unique site visitors)**<br>Requires counting unique visitors to the first page of the survey, divided by the number of unique site visitors (not page views!). It is not unusual to have view rates of less than 0.1% if the survey is voluntary.	N/A
22		**Participation rate**<br>**(Ratio of unique visitors who agreed to participate/unique first survey page visitors)**<br>Count the unique number of people who filled in the first survey page (or agreed to participate, for example by checking a checkbox), divided by visitors who visit the first page of the survey (or the informed consents page, if present). This can also be called 'recruitment' rate.	N/A
23		**Completion rate**<br>**(Ratio of users who finished the survey/users who agreed to participate)**<br>The number of people submitting the last questionnaire page, divided by the number of people who agreed to participate (or submitted the first survey page). This is only relevant if there is a separate 'informed consent' page or if the survey goes over several pages. This is a measure for attrition. Note that 'completion' can involve leaving questionnaire items blank. This is not a measure for how completely questionnaires were filled in. (If you need a measure for this use the word 'completeness rate'.)	N/A
24	Preventing multiple entries from the same individual	**Cookies used**<br>Indicate whether cookies were used to assign a unique user identifier to each client computer. If so, mention the page on which the cookie was set and read, and how long the cookie was valid. Were duplicate entries avoided by preventing users access to the survey twice; or were duplicate database entries having the same user ID eliminated before analysis? If the latter, which entries were kept for analysis (eg the first entry or the most recent)?	N/A
25		**IP check**<br>Indicate whether the IP address of the client computer was used to identify potential duplicate entries from the same user. If so, mention the period of time for which no two entries from the same IP address were allowed (eg 24 hours). Were duplicate entries avoided by preventing users with the same IP address access to the survey twice; or were duplicate database entries having the same IP address within a given period of time eliminated before analysis? If the latter, which entries were kept for analysis (eg the first entry or the most recent)?	8
26		**Log file analysis**<br>Indicate whether other techniques to analyse the log file for identification of multiple entries were used. If so, please describe.	N/A
27		**Registration**<br>In 'closed' (non-open) surveys, users need to login first and it is easier to prevent duplicate entries from the same user. Describe how this was done. For example, was the survey never displayed a second time once the user had filled	N/A

For peer review only

		it in, or was the username stored together with the survey results and later eliminated? If the latter, which entries were kept for analysis (eg the first entry or the most recent)?	
28	Analysis	**Handling of incomplete questionnaires**<br>Were only completed questionnaires analysed? Were questionnaires which terminated early (where, for example, users did not go through all questionnaire pages) also analysed?	9
29		**Questionnaires submitted with an atypical timestamp**<br>Some investigators may measure the time people needed to fill in a questionnaire and exclude questionnaires that were submitted too soon. Specify the timeframe that was used as a cut-off point and describe how this point was determined.	N/A
30		**Statistical correction**<br>Indicate whether any methods such as weighting of items or propensity scores have been used to adjust for the non-representative sample; if so, please describe the methods.	N/A

For peer review only

BMJ Open

Consumer understanding of terms used in imaging reports requested for low back pain: a cross-sectional survey

Journal:	*BMJ Open*
Manuscript ID	bmjopen-2021-049938.R1
Article Type:	Original research
Date Submitted by the Author:	27-May-2021
Complete List of Authors:	Farmer, Caitlin; Monash University, Department of Epidemiology and Preventive Medicine, School of Public Health and Preventive Medicine; Monash University, Department of Clinical Epidemiology, Cabrini Health<br>O'Connor, Denise; Monash University, Department of Epidemiology and Preventive Medicine, School of Public Health and Preventive Medicine; Monash University, Department of Clinical Epidemiology, Cabrini Health<br>Lee, Hopin; University of Oxford, Centre for Statistics in Medicine, Rehabilitation Research in Oxford, Nuffield Department of Orthopaedics Rheumatology and Musculoskeletal Sciences (NDORMS), ; The University of Newcastle, School of Medicine and Public Health<br>McCaffery, Kirsten; The University of Sydney, Sydney Health Literacy Lab, School of Public Health<br>Maher, Christopher; The University of Sydney, School of Public Health; The University of Sydney Institute for Musculoskeletal Health<br>Newell, Dave; AECC University College<br>Cashin, Aidan; Neuroscience Research Australia, Centre for Pain IMPACT; University of New South Wales, Prince of Wales Clinical School<br>Byfield, David; University of South Wales Faculty of Life Sciences and Education<br>Jarvik, Jeffrey; University of Washington, Departments of Radiology, Neurological Surgery and Health Services, School of Medicine; University of Washington, UW Clinical Learning, Evidence And Research (CLEAR) Center for Musculoskeletal Disorders<br>Buchbinder, Rachelle; Monash University Faculty of Medicine Nursing and Health Sciences, Dept of Epidemiology and Preventive Medicine, School of Public Health and Preventive Medicine; Monash University, Department of Clinical Epidemiology, Cabrini Health
**Primary Subject Heading**:	Radiology and imaging
Secondary Subject Heading:	Public health
Keywords:	Diagnostic radiology < RADIOLOGY & IMAGING, Back pain < ORTHOPAEDIC & TRAUMA SURGERY, Musculoskeletal disorders < ORTHOPAEDIC & TRAUMA SURGERY

SCHOLARONE™ Manuscripts

I, the Submitting Author has the right to grant and does grant on behalf of all authors of the Work (as defined in the below author licence), an exclusive licence and/or a non-exclusive licence for contributions from authors who are: i) UK Crown employees; ii) where BMJ has agreed a CC-BY licence shall apply, and/or iii) in accordance with the terms applicable for US Federal Government officers or employees acting as part of their official duties; on a worldwide, perpetual, irrevocable, royalty-free basis to BMJ Publishing Group Ltd ("BMJ") its licensees and where the relevant Journal is co-owned by BMJ to the co-owners of the Journal, to publish the Work in this journal and any other BMJ products and to exploit all rights, as set out in our licence.

The Submitting Author accepts and understands that any supply made under these terms is made by BMJ to the Submitting Author unless you are acting as an employee on behalf of your employer or a postgraduate student of an affiliated institution which is paying any applicable article publishing charge ("APC") for Open Access articles. Where the Submitting Author wishes to make the Work available on an Open Access basis (and intends to pay the relevant APC), the terms of reuse of such Open Access shall be governed by a Creative Commons licence – details of these licences and which Creative Commons licence will apply to this Work are set out in our licence referred to above.

Other than as permitted in any relevant BMJ Author's Self Archiving Policies, I confirm this Work has not been accepted for publication elsewhere, is not being considered for publication elsewhere and does not duplicate material already published. I confirm all authors consent to publication of this Work and authorise the granting of this licence.

For peer review only

Consumer understanding of terms used in imaging reports requested for low back pain: a cross-sectional survey

Caitlin Farmer[1,2], Denise O'Connor[1,2], Hopin Lee[3,4], Kirsten McCaffery[5], Christopher Maher[6,7], Dave Newell[8], Aidan Cashin[9,10], David Byfield[11], Jeffrey G. Jarvik[12,13], Rachelle Buchbinder[1,2]

[1]Department of Epidemiology and Preventive Medicine, School of Public Health and Preventive Medicine, Monash University, Melbourne, Australia.

[2]Monash Department of Clinical Epidemiology, Cabrini Health, Melbourne, Australia.

[3]Centre for Statistics in Medicine, Rehabilitation Research in Oxford, Nuffield Department of Orthopaedics Rheumatology and Musculoskeletal Sciences (NDORMS), University of Oxford, Oxford, United Kingdom.

[4]School of Medicine and Public Health, University of Newcastle, Newcastle, Australia.

[5]Sydney Health Literacy Lab, Wiser Healthcare, Sydney School of Public Health, Faculty of Medicine and Health, University of Sydney, Sydney, Australia

[6]School of Public Health, University of Sydney, Sydney, Australia.

[7]Institute for Musculoskeletal Health, Sydney, Australia.

[8]AECC University College, Dorset, United Kingdom.

[9]Centre for Pain IMPACT, Neuroscience Research Australia, Sydney, Australia.

[10]Prince of Wales Clinical School, Faculty of Medicine, University of New South Wales, Sydney, Australia.

[11]Faculty of Life Sciences and Education University of South Wales, Treforest, United Kingdom.

[12]Departments of Radiology, Neurological Surgery and Health Services, School of Medicine, University of Washington, United States.

[13]UW Clinical Learning, Evidence And Research (CLEAR) Center for Musculoskeletal Disorders, Seattle, WA, United States.

Corresponding author:

Caitlin Farmer

Monash Department of Clinical Epidemiology

4 Drysdale St

Malvern VIC 3144

Australia

caitlin.farmer1@monash.edu

Word Count: 3,548 excluding title page, abstract, tables and references

For peer review only

ABSTRACT

Objectives: To investigate 1) self-reported societal comprehension of common and usually non-serious terms found in lumbar spine imaging reports and 2) its relationship to perceived seriousness, likely persistence of low back pain (LBP), fear of movement, back beliefs and history and intensity of low back pain.

Design: Cross-sectional online survey of the general public.

Setting: Five English-speaking countries: United Kingdom, United States, Canada, New Zealand, Australia.

Participants: Adults (age > 18 years) with or without a history of LBP recruited in April 2019 with quotas for country, age and gender.

Primary and secondary outcome measures: Self-reported understanding of 14 terms (annular fissure, disc bulge, disc degeneration, disc extrusion, disc height loss, disc protrusion, disc signal loss, facet joint degeneration, high intensity zone, mild canal stenosis, modic changes, nerve root contact, spondylolisthesis and spondylosis) commonly found in lumbar spine imaging reports. For each term we also elicited worry about its seriousness, and whether its presence would indicate pain persistence and prompt fear of movement.

Results: From 774 responses we included 677 (87.5%) with complete and valid responses. 577 (85%) participants had a current or past history of LBP of whom 251 (44%) had received lumbar spine imaging. Self-reported understanding of all terms was poor. At best, 235 (35%) reported understanding the term 'disc degeneration', while only 71 (10.5%) reported understanding the term 'modic changes'. For all terms, a moderate to large proportion of participants (range 59-71%), considered they indicated a serious back problem, that pain might persist (range 52-71%) and they would be fearful of movement (range 42-57%).

Conclusion: Common and usually non-serious terms in lumbar spine imaging reports are poorly understood by the general population and may contribute to the burden of LBP.

Trial registration: This study was prospectively registered with the Australian & New Zealand Clinical Trials Registry (12619000545167).

STRENGTHS AND LIMITATIONS OF THIS STUDY

- This is the first study to directly measure societal self-reported understanding of 14 commonly used terms found in lumbar spine imaging reports and its relationship to worry regarding the seriousness and persistence of low back pain and being fearful of movement.
- Participants were a large representative sample from five high-income English-speaking countries with quotas for age and gender suggesting our results are likely generalisable to those settings but may or may not be generalisable to other settings.
- Self-reported understanding may not truly reflect a person's understanding of the terms, and the use of a survey sampling company may have introduced some bias.
- Although all terms that we investigated are findings that commonly exist in the asymptomatic population, some are more common in people with low back pain, and this may have influenced participants' responses.

review only

INTRODUCTION

Low back pain (LBP) is a common, costly problem and a significant contributor to global disability.(1) Most LBP is labelled 'non-specific', reflecting an inability to identify a specific cause in most cases.(2) In the absence of features suggestive of a serious cause, clinical practice guidelines consistently recommend against any form of lumbar spine imaging as it is unlikely to provide meaningful information about the cause of pain or guide treatment.(3-5) Imaging often reveals age-related changes such as disc degeneration, disc bulge and facet joint degeneration that are also common in asymptomatic people.(6)

Lumbar spine imaging also carries risk of iatrogenic harm. This includes not only exposure to ionising radiation with plain radiographs and CT scans, but the way findings are described may lead to clinician and patient anxiety about perceived seriousness, prompt further unnecessary tests, unwarranted diagnostic labels and unnecessary treatment.(7, 8)

While few published studies have investigated consumer understanding and views regarding terminology in lumbar spine imaging findings in people with LBP, several studies have investigated these issues for other conditions.(9-14) A systematic review investigating how different terms for various conditions influence management preferences found that use of more medicalised or precise terms generally results in greater anxiety and perceived severity, and preferences towards more invasive care.(15) Ambiguous terminology for incidental low-risk liver lesions reported in abdominal CT reports was found to increase patient and referrer anxiety and a greater likelihood of follow-up testing.(16) In contrast, simplified upper limb MRI reports using more understandable and less 'emotive' language (e.g., 'expected age-related changes' rather than 'hypertrophic degenerative changes') have been found to reduce worry, improve comprehension and help patients feel more in control of their health.(17)

For peer review only

As patients are increasingly accessing their imaging reports, it is important to understand how commonly reported terms are interpreted by patients. The primary aim of this study was to investigate self-reported understanding of terms that are both commonly found in lumbar spine imaging reports and are usually of little clinical relevance. We also investigated the relationship between self-reported understanding of these terms and their perceived seriousness, perceptions about the likelihood of pain persistence and whether they would result in being fearful of movement. Our secondary aim was to describe the association between self-reported understanding of the terms and back beliefs, and history and intensity of LBP. We also elicited views about access to imaging reports and whether they should be understandable to patients and include context.

METHODS

Design

This study was a cross-sectional online survey of a random sample of the general public in five English-speaking countries: Australia, New Zealand, the United States, United Kingdom, Canada. It was conducted and reported in accordance with the Checklist for Reporting Results of Internet E-Surveys (CHERRIES) guideline (18).

Participants and recruitment

Participants were recruited in April 2019 via an online survey sampling company (Dynata). Dynata is a data company that maintains a research and marketing database. It recruits participants via email based on the requirements of the research involved. Upon survey completion, Dynata provides participant incentives that allow redemption of items such as gift cards and contributions to charity. English-speaking adults aged 18 and over with or without a

For peer review only

current or past history of back pain were recruited via email with quotas established for country, age and gender to ensure a representative sample.

The survey was conducted in Qualtrics and no identifying information was provided to the research team.

Survey instrument

Pilot testing conducted in a convenience sample of ten members of the general public resulted in minor changes. The survey included nine demographic questions (age in years, gender (female/male/other/prefer not to say), education level (finished high school/technical/trade certificate or diploma, university (Bachelor), post-graduate university qualifications), employment status (full time/part time/unemployed/student/home duties/retired (not due to ill health)/retired (due to ill health)/other), health care provider for people with LBP (yes/no), LBP history (current, past, never) and for those with a history of LBP we asked about average pain intensity (measured on a 0 to 100 point VAS where higher scores indicate greater pain), LBP imaging (Yes/No/Don't Know) and treatment (surgery and injections).

Four statements were presented for each of 14 radiology terms (annular fissure, disc bulge, disc degeneration, disc extrusion, disc height loss, disc protrusion, disc signal loss, facet joint degeneration, high intensity zone, mild canal stenosis, modic changes, nerve root contact, spondylolisthesis and spondylosis). These terms were chosen on the basis that they are commonly found in lumbar imaging reports of people with LBP but are also common findings in asymptomatic populations and are usually considered benign or of unclear clinical significance.(6, 19, 20) For each question, order of presentation of the terms was randomised to reduce order effect bias.

For peer review only - http://bmjopen.bmj.com/site/about/guidelines.xhtml

For each item, we elicited the participant's level of agreement on a 5-point Likert scale (1=Completely disagree, 2=Somewhat disagree, 3=Neither agree nor disagree, 4=Somewhat agree, 5=Completely agree) to the following four statements:

1. I would need to look this term up to know what it means (Understanding)
2. I would be worried there is a serious problem with my back (Seriousness)
3. I would be worried my pain isn't going to get any better (Persistence)
4. I would be afraid to move my back in case I did more damage (Fear of movement)

Items 2 and 3 were derived from questionnaires for musculoskeletal conditions or LBP that assess perceived seriousness or likely persistence of pain.(21-23) For example the third item is similar to items in the STarT Back Tool ('I feel my back pain is terrible and it's never going to get any better'),(24) Pain Catastrophizing Scale ('It's terrible and I think it's never going to get any better')(25) and the Back-PAQ ('There is a high chance that an episode of back pain will not resolve').(22)

We also elicited opinions (yes/no/don't know) about whether people should have access to their imaging reports; whether imaging reports should be written so they can be understood by lay people; and whether including epidemiological information about common findings in people without back pain would be helpful in interpreting results.

The Back Beliefs Questionnaire (BBQ) is a 14 item tool that measures beliefs about back pain.(26) Responses to each item are measured on a 5-point Likert agreement scale (completely disagree to completely agree). The BBQ is scored from nine of the 14 items with higher scores indicating more positive beliefs (score range 9 to 45).

For peer review only - http://bmjopen.bmj.com/site/about/guidelines.xhtml

Sample size and analysis

A minimum sample size of 590 was required to detect 70% ± 10% prevalence of not understanding a term. Assuming 10% drop out or non-completion of surveys, our target was 650 participants. This sample size also provided enough power to investigate associations between variables.

Raw data files were reviewed for evidence of duplicate answers including duplicate internet protocol (IP) addresses prior to inclusion in the analysis. Descriptive statistics were used to summarise the participant characteristics, responses to statements about radiological terms, back beliefs and opinions regarding imaging reports. Data were excluded for any participant who provided the same answer for every item of the BBQ as this type of response is invalid and likely indicates that the questionnaire responses for other items were also invalid.

Responses of either completely disagree or somewhat disagree with the statement 'I would need to look this up to know what it means' were considered to indicate self-reported understanding of a term.

Responses for each item were dichotomised with ‘completely disagree’ and ‘somewhat disagree’ coded as zero and ‘neither agree nor disagree’/’somewhat agree’/’completely agree’ coded as one. For self-reported understanding, we combined a neutral response (neither agree nor disagree) with somewhat or completely agree as we considered this response more likely indicated that a respondent didn’t understand a term. For items regarding worry about seriousness, persistence of pain and fear of movement, we similarly combined a neutral

For peer review only

response with somewhat or completely agree with these statements (ie, worried about seriousness, pain persistence or being fearful of movement).

Dichotomised scores were summed into total response numbers for each participant for the 14 radiology terms ranging from 0 to 14. For the item about understanding, a score of 0 indicates that respondents reported that they would not need to look up any of the 14 terms (high level of understanding), while a score of 14 indicates they would need to look up all terms (low understanding). For items regarding worry about seriousness, persistence of pain and fear of movement, a score of 0 indicates that none of the terms elicited worry about seriousness, persistence of pain and/or movement, while a score of 14 indicates that all terms elicited concerns about seriousness, persistence of pain and/or movement. Following distribution assessment of scores, they were reversed.

After assessment of best fit via the 'nbvargr' Stata command, multivariate negative binomial regression was used to investigate the association between self-reported understanding of terms and perceived seriousness, risk of persistence and fear of movement, and their association with participant demographics, history of LBP and BBQ score. Spearman's rank correlation was used to investigate the correlation between responses to the four statements regarding the terms.

Ethics

This project was approved by the Monash University Human Research Ethics Committee (ID 13896). Survey completion was considered consent to participate and participants were able to review their answers prior to submission.

For peer review only - http://bmjopen.bmj.com/site/about/guidelines.xhtml

Patient and public involvement

Consumer feedback about questionnaire burden and time requirements was incorporated into survey design. Survey participants were advised how to access results of the study in the accompanying explanatory statement.

RESULTS

Of 774 responses, we excluded 97 (12.5%) due to incomplete data (n=35, 4.5%), invalid BBQ responses (n=59, 7.6%) and age given as under 18 years (n=3, 0.4%). No duplicate responses were identified. We were unable to calculate a response rate as Dynata were unable to provide precise data about how often the survey was available to users via either email or their dashboard.

Responses from 677 participants (52% female) were analysed (Table 1). There were no significant between-country differences regarding demographic characteristics. 577 participants (85%) reported past or current LBP, and 279 (41%) reported having LBP at the time of survey completion. The mean (SD) BBQ score for the whole cohort was 25.0 (6.4). Pain intensity was higher in those who reported current compared with past LBP (mean (SD): 54.7 (21.3), n=279, versus 44.2 (22.1), n=298, respectively, $p<0.001$). Of those with a history of LBP, 251 (44%) reported receipt of lumbar spine imaging, 81 (14%) reported receipt of spine injection/s and 27 (5%) reported undergoing spine surgery.

For peer review only - http://bmjopen.bmj.com/site/about/guidelines.xhtml

Table 1: Participant demographics and back pain history and beliefs by country and overall

		USA (n=139)	Australia (n=138)	NZ (n=135)	UK (n=132)	Canada (n=133)	TOTAL (n=677)
		Mean (SD)	*Mean (SD)*	*Mean (SD)*	*Mean (SD)*	*Mean (SD)*	*Mean (SD)*
Age, years		45.4 (16.0)	45.0 (17.0)	45.9 (17.6)	47.6 (17.0)	45.4 (15.5)	45.8 (16.4)
Back pain intensity most recent episode* (0-100 scale, higher score indicates greater pain)	Current LBP	49.7 (22.4)	58.2 (20.2)	55.0 (21.7)	58.1 (22.0)	51.9 (20.2)	54.7 (21.3)
	Previous LBP	40.0 (19.7)	48.4 (22.6)	44.2 (21.3)	45.4 (24.4)	44.0 (22.6)	44.2 (22.1)
BBQ score (9-45 scale, higher score indicates better back beliefs)		25.7 (6.4)	24.3 (6.5)	25.8 (6.6)	24.6 (6.3)	24.4 (6.1)	25.0 (6.4)
		N (%)	*N (%)*	*N (%)*	*N (%)*	*N (%)*	*N (%)*
Female		75 (54)	73 (53)	70 (52)	67 (51)	69 (52)	354 (52)
University education		69 (50)	51 (37)	63 (47)	59 (45)	57 (43)	299 (44)
Employed full or part time		71 (51)	80 (58)	77 (57)	73 (55)	80 (60)	381 (56)
Worked as healthcare professional#		10 (7)	4 (3)	4 (3)	5 (4)	4 (3)	27 (4)
Ever had low back pain		116 (83)	119 (86)	114 (84)	113 (86)	115 (86)	577 (85)
Current low back pain		54 (38)	68 (49)	45 (33)	52 (39)	60 (45)	279 (41)
Had lumbar spine imaging*		60 (52)	60 (50)	40 (35)	38 (34)	53 (46)	251 (44)
Had lumbar spine injection*		17 (15)	18 (15)	16 (14)	21 (19)	9 (8)	81 (14)
Had lumbar spine surgery*		6 (5)	4 (3)	7 (6)	6 (5)	4 (4)	27 (5)

*Only for participants who responded 'yes' to current or previous history of low back pain
#Health professionals included 9 nurses, 4 doctors (1 GP), 3 health care assistants, 2 pharmacists, an occupational therapist, pharmacy technician, physical therapist, physician assistant, prescription manager, psychologist, respiratory therapist and a 'therapist'.
Abbreviations: USA – United States of America, NZ – New Zealand, UK – United Kingdom, SD – Standard Deviation, BBQ – Back beliefs questionnaire, LBP – Low back pain

For peer review only - http://bmjopen.bmj.com/site/about/guidelines.xhtml

Participant views regarding terms

Most participants (n=598, 88%) somewhat or completely agreed they would need to look up at least one of the terms to know what it means, while only 3% (n=17) somewhat or completely disagreed with this statement for all terms (Figure 1). Overall, 41 (6%) participants provided neutral answers for all comprehension questions (i.e., neither agree nor disagree).

The proportion who reported understanding the meaning of terms ranged from 35% (n=235) for the term 'disc degeneration' to 10.5% (n=71) for 'modic changes' (Figure 1a). The same proportion (88%) somewhat or completely agreed they would be worried about a serious problem with their back for at least one term and only 3% (n=17) disagreed with this statement for all terms (Figure 1b). Three terms, 'disc extrusion', 'disc degeneration' and 'facet joint degeneration', elucidated the most worry about seriousness (71% of participants). Worry regarding seriousness varied only a small amount between terms, with the number of participants disagreeing with the statement varying between only 7% and 11% for all terms.

For all terms, over half the participants were worried that back pain would persist if the imaging finding was present (Figure 1c). The least number of participants (51%) reported worry regarding persistence for the term 'modic changes', while 'disc degeneration' was the term which caused worry in the greatest percent of participants (71%). In total 84% of participants agreed they would be worried regarding persistence for at least one term, and 4% of participants disagreed with the statement for all terms.

For many imaging findings, participants also frequently reported they would be afraid to move their back. 74% agreed with this statement for at least one term and only 8% disagreed

For peer review only - http://bmjopen.bmj.com/site/about/guidelines.xhtml

with this statement for all terms (Figure 1d). The greatest number of participants agreed with this statement for the term ‘disc bulge’ (57%), and 56% of participants also agreed with this statement for the terms ‘disc degeneration’, ‘disc protrusion’, ‘nerve root contact’ and ‘disc extrusion’. ‘Modic changes’ and ‘mild canal stenosis’ were the terms associated with least worry regarding movement (42% agreed with the statement).

Associations between self-reported understanding of terms and perceived seriousness, risk of pain persistence and fear of movement

There was moderate correlation between understanding of terms and worry about seriousness, pain persistence and fear of movement (Spearman’s rho 0.48, p<0.001, 0.39, p<0.001 and 0.38, p<0.001 respectively) (Table 2). Strong associations were present between the responses regarding worry about seriousness and pain persistence (Spearman’s rho 0.63, p<0.001) and pain persistence and fear of movement (0.51, p<0.001).

Table 2: Association between the self-reported understanding of the 14 terms overall and perceived seriousness, pain persistence and fear of movement, Spearman’s rho correlation*

	Understanding	Seriousness	Pain persistence	Fear of movement
**Understanding**	1			
**Seriousness**	0.48	1		
**Persistence**	0.39	0.63	1	
**Fear of movement**	0.38	0.42	0.51	1

**All p values <0.001.*
Values between 0.10 and 0.29 represent a weak association, 0.30-0.49 moderate and 0.5 and above a strong association.

For peer review only - http://bmjopen.bmj.com/site/about/guidelines.xhtml

Table 3: Association between self-reported understanding of the 14 terms, perceived seriousness, pain persistence and fear of movement and demographic details, history of back pain and back beliefs

	**Understanding**	**Seriousness**	**Pain persistence**	**Fear of movement**
	Regression coefficient (95% Confidence Interval)			
Education (REF - High school)				
Trade/Diploma	**0.32 (0.07, 0.57)***	-0.27 (-0.57, 0.03)	**-0.73 (-1.02 to -0.43)*****	**-0.37 (-0.63, -0.11)****
Bachelor	**0.34 (0.12, 0.57)****	**- 0.35 (-0.62, -0.09)****	**-0.38 (-0.65 to -0.11)****	**-0.44 (-0.68 to -0.20)*****
Postgraduate qualifications	**0.51 (0.23, 0.80)*****	**-0.69 (-1.01, -0.36)*****	**-0.98 (-1.31, -0.66)*****	**-0.61 (-0.91, -0.32)*****
LBP History (REF - Never)				
Previous LBP	0.20 (-0.08, 0.48)	0.27 (-0.04, 0.57)	0.01 (-0.30 to 0.32)	**-0.32 (-0.61, -0.02)***
Current LBP	**0.42 (0.14, 0.70)****	0.10 (-0.22, 0.42)	-0.13 (-0.45, 0.19)	**-0.61 (-0.91, -0.31)*****
BBQ score	**0.02 (0.004, 0.03)***	**-0.03 (-0.05 to -0.02)*****	**-0.06 (-0.08 to -0.04)*****	**-0.07 (-0.08, -0.05)*****
Age, years	0.002 (-0.004, 0.01)	**0.02 (0.01, 0.03)*****	**0.02 (0.02, 0.03)*****	**-0.007 (-0.01, -0.001)***
Gender (REF - Female)				
Male	0.06 (-0.12, 0.24)	**-0.31 (-0.52, -0.09)****	-0.19 (-0.40, 0.03)	**-0.21 (-0.40, -0.02)***

Note: Multivariate negative binomial regression results – bold indicates statistically significant, * $p < 0.05$, **$p < 0.01$, ***$p < 0.001$. Results rounded to two decimal points or first meaningful decimal point. REF = Reference category, LBP = Low Back Pain, BBQ = Back Beliefs Questionnaire

Continuous variables (BBQ score and age): For understanding, a positive regression coefficient indicates greater self-reported understanding is associated with better back beliefs (higher BBQ score) and increased age. For seriousness, pain persistence and fear of movement, a positive regression coefficient indicates greater worry/concern is associated with poorer back beliefs (lower BBQ score) and increased age.

Categorical variables: For understanding, a positive regression coefficient indicates increased self-reported understanding compared with the reference category. For seriousness, pain persistence and fear of movement, a positive regression coefficient indicates greater worry/concern compared with the reference variable.

For peer review only - http://bmjopen.bmj.com/site/about/guidelines.xhtml

Associations with demographic features, history of back pain and back beliefs

Results of the regression analysis are presented in Table 3. Higher education level was associated with greater self-reported understanding of terms, and less concern about seriousness, pain persistence and being fearful of movement. Participants with current LBP had better understanding and less fear of movement compared with those without a history of LBP. More positive back beliefs (higher BBQ score) were associated with greater self-reported understanding of terms and less worry regarding seriousness, persistence and movement. Older age was associated with greater worry about seriousness and pain persistence but less fear of movement. Male gender was associated with less worry about seriousness and movement compared with female gender.

Participants views about imaging reports

The majority of participants (n=605, 89%) agreed that people with LBP should have access to their radiology report, that it should be written in a way to facilitate lay understanding (n=586, 87%) and it would be useful to include information about the prevalence of common imaging findings in people without back pain (n=587, 87%).

Discussion

Our study found evidence of poor self-reported understanding of terms commonly used to describe usually benign findings reported in lumbar spine imaging reports. Most participants had concerns about their seriousness and associated the terms with persistence of pain and/or need to avoid (or be fearful of) movement. Higher education, more positive back beliefs and current LBP were all associated with greater self-reported understanding of the terms, while people with higher education and more positive back beliefs were also less concerned about seriousness, pain persistence and being fearful of movement.

For peer review only - http://bmjopen.bmj.com/site/about/guidelines.xhtml

Our findings, consistent with other literature that has found that language matters, (15, 16) provide support for changing how these findings are reported. Recent online scenario-based randomised experiments that provided imaging reports for 'virtual patients' with LBP found that altering the language and/or providing epidemiologic information improved perceptions regarding LBP compared with provision of a standard report.(27, 28) This now requires confirmation in randomised trials of real patients.

The radiology report may play a role in mediating the relationship between imaging and overtreatment in LBP, (7, 29) and providing contextual information has been reported to reduce unnecessary repeat imaging, opioid prescription and specialist referral.(30, 31) Trials that have assessed the provision of epidemiologic data or additional educational messaging, (32-36) including for LBP, (20, 36, 37) have been designed to improve referrer knowledge and behaviour, but none have assessed patient knowledge or other outcomes. Our study provides evidence that commonly identified and reported terms in radiology reports are also associated with misplaced patient concerns about their implications, which may also influence their healthcare decision-making.

Those with current LBP had poorer back beliefs compared to those without a history of LBP or past LBP. This is consistent with previous studies that have found greater pessimism regarding recovery among those with current symptoms.(26, 38-41) This may also explain their increased expectation and wish for further tests and treatment.(42)

For peer review only - http://bmjopen.bmj.com/site/about/guidelines.xhtml

Strengths and limitations

To our knowledge this is the first study to measure societal self-reported understanding of commonly used terms in lumbar spine imaging reports and their relationship to other factors such as perceived seriousness, persistence of pain and fear of movement. Although we were unable to calculate a response rate, it is likely our results are generalisable to the five English-speaking countries we included as we used age and gender quotas to reflect the make-up of each country. Additionally, the proportion of participants with a life-time history of LBP is consistent with other population-based studies, (38, 40), although the prevalence of current low back pain was slightly higher which may indicate greater interest in completing a survey about LBP. BBQ scores of our participants were also similar to other population-based cohorts.(38, 40, 41, 43, 44)

On the other hand, we used a survey sampling company, which may have introduced participation bias as participants receive rewards albeit very small ones. In addition, people who are online and familiar with computers and online questionnaires may differ from the general population. Our sample included a small number of health professionals with diverse roles and we therefore think that excluding them from our analysis would have been unlikely to change our results. We also did not consider that any meaningful conclusions could be drawn from analysing this group separately. Our study was appropriately powered and randomisation of the terms for each participant minimised any order effect bias. Where possible we adapted our survey items from other tools with established validity and reliability and the survey was piloted prior to use.

Survey measurement of public understanding of medical terms is challenging. Asking respondents whether they would need to look the term up to know what it means could be considered an even more indirect measure of self-reported understanding. As well, self-reported understanding of a medical term may not be a true reflection of participants' true understanding of terms and our study may have over or underestimated true understanding. Similarly, we did not include a measure of health literacy in our survey and we therefore do not know how differing health literacy skills may have influenced our findings. A large proportion of answers relating to the items were neutral (i.e., neither agree nor disagree), however only 19 (2.8%) of participants provided neutral answers for all items and they had a wide range of BBQ scores ranging from 15-45 suggesting that their neutral answers were likely genuine.

All of the imaging findings we assessed frequently exist in the asymptomatic population and therefore may or may not have relevance in an individual case. However, some findings such as disc bulge, disc extrusion, Modic changes, disc protrusion and disc degeneration, occur more commonly in symptomatic populations.(19) Participants' past experiences with LBP and/or imaging of the lumbar spine and these terms may have influenced their responses.

Implications for practice

All terms we investigated elicited concerns that were likely unwarranted. Combined with our finding that most participants would like access to their reports, more consideration is needed about how to report findings in lumbar imaging reports in people with LBP. Our finding that respondents wanted reports to be understandable to the lay person is also consistent with the results of other studies.(45, 46)

For peer review only

Although clinicians may have reservations about allowing patients direct access to their medical information,(47) there is already increased availability and use of patient portals (48) and in many health systems this is standard practice.(49) In organisations where this has occurred, radiology reports are one of the most common items reviewed by patients accessing their information. (50) A recent addition to patient portals has been a lay-language glossary (51) however with a current focus on defining anatomical structures it may not reduce patient concern regarding benign findings. Any changes to patient interpretation of radiology reports as a result of using the glossary have not been evaluated.

Moreover, there is clear evidence that the low back pain beliefs and attitudes of health care professionals have an impact on patient beliefs and affect their clinical care. (52) A recent study found that the way lumbar spine imaging is reported can influence the beliefs of clinicians regarding the severity of the condition and their approach to management, including their views on whether surgery may be required. (53) Along with more in depth exploration of the impact of patient attitudes and beliefs regarding radiology findings, identifying and addressing the clinician factors that trigger low value care based upon imaging findings are important areas for future research.

It is likely that our results may be generalisable to other regional musculoskeletal conditions where imaging also often reveals age-related changes that are common in asymptomatic people. These changes include disc bulges in the cervical spine, (54) meniscal tears in the knee (55) and supraspinatus tendinosis in the shoulder. (56) Our study could be replicated for these conditions to determine if our study findings are also applicable to them.

For peer review only - http://bmjopen.bmj.com/site/about/guidelines.xhtml

There is now both imperative and empirical data indicating the importance of accurately and clearly portraying the significance of imaging findings in terms that are understandable to both clinicians and patients.

CONCLUSION

Common and usually non-serious terms in lumbar spine imaging reports are poorly understood by the general population and may contribute to the burden of LBP. Incorporating clear explanations about the implications of these findings may reduce unwarranted anxiety and reduce low-value care.

For peer review only

Contributorship statement

CF contributed to the conception and design of this study, the acquisition, analysis and interpretation of the data, drafting and revising the work. DO contributed to the design of this study, the acquisition, analysis and interpretation of the data, drafting and revising the work. HL contributed to the conception and design of this study, the analysis and interpretation of the data and revising the work critically for content. KM contributed to the conception and design of this study, the interpretation of the data and revising the work critically for content. CM contributed to the conception and design of this study, the analysis and interpretation of the data and revising the work critically for content. DN contributed to the conception and design of this study, the interpretation of the data and revising the work critically for content. AC contributed to the conception and design of this study, the interpretation of the data and revising the work critically for content. DB contributed to the conception and design of this study, the interpretation of the data and revising the work critically for content. JJ contributed to the conception and design of this study, the interpretation of the data and revising the work critically for content. RB contributed to the conception and design of this study, the acquisition, analysis and interpretation of the data, drafting and revising the work. All authors approved the final version and agree to be accountable for all aspects of this study.

Acknowledgement

We acknowledge and thank Alex Gorelik for her assistance with data analysis for this study.

Competing interests statement

No competing interests relating to this publication have been declared. CM and RB have received grants from numerous government and not-for-profit agencies; their expenses have been covered by professional associations hosting conferences where they were speakers, and they were both investigators for a trial that received heat wraps from Flexeze at no cost.

For peer review only - http://bmjopen.bmj.com/site/about/guidelines.xhtml

Funding statement

DO is supported by an Australian National Health and Medical Research Council (NHMRC) Translating Research into Practice Fellowship (APP1168749). HL is funded by an NHMRC (grant no. APP1126767); receives project funding from the Berkeley Initiative for Transparency in the Social Sciences, a program of the Center for Effective Global Action (CEGA), with support from the Laura and John Arnold Foundation. He is also supported by funding from the National Institute for Health Research (NIHR) Collaboration for Leadership in Applied Health Research and Care Oxford at Oxford Health NHS Foundation Trust. JGJ is supported by funding from the US National Institute of Health/NIAMS (P30AR072572). CM is supported by an NHMRC Principal Research Fellowship (APP1103022). RB is supported by an NHMRC Senior Principal Research Fellowship (APP1082138).

Data sharing statement

The study protocol and deidentified participant data is available upon reasonable request to the corresponding author (caitlin.farmer1@monash.edu).

Ethical statement

This project was approved by the Monash University Human Research Ethics Committee (ID 13896). Completion of the survey was considered consent to participate.

Figure legend/caption

Figure 1: Responses to questions regarding level of understanding, seriousness, persistence of pain and fear of movement for the 14 radiology terms

For peer review only

Note: For each term, we asked four questions with a common opening ('If this term was in my report I would...') to evaluate a) understanding ('....need to look this term up to know what it means'); b) worry about seriousness ('...be worried there is a serious problem with my back'); c) worry about persistence ('...be worried my pain would persist') and d) fear of movement ('...be afraid to move in case I did more damage').

For peer review only

References

1. Hartvigsen J, Hancock MJ, Kongsted A, et al. What low back pain is and why we need to pay attention. *The Lancet*. 2018. doi:10.1016/s0140-6736(18)30480-x

2. Maher C, Underwood M, Buchbinder R. Non-specific low back pain. *The Lancet*. 2017;389(10070):736-47. doi:10.1016/s0140-6736(16)30970-9

3. National Institute for Health and Care Excellence: Clinical Guidelines. Low Back Pain and Sciatica in Over 16s: Assessment and Management. London: National Institute for Health and Care Excellence (UK); 2016.

4. Chou R, Qaseem A, Snow V, et al. Diagnosis and treatment of low back pain: A joint clinical practice guideline from the american college of physicians and the american pain society. *Annals of Internal Medicine*. 2007;147(7):478-91. doi:10.7326/0003-4819-147-7-200710020-00006

5. Oliveira CB, Maher CG, Pinto RZ, et al. Clinical practice guidelines for the management of non-specific low back pain in primary care: an updated overview. *European Spine Journal*. 2018;27(11):2791-803. doi:10.1007/s00586-018-5673-2

6. Brinjikji W, Luetmer PH, Comstock B, et al. Systematic literature review of imaging features of spinal degeneration in asymptomatic populations. *AJNR Am J Neuroradiol*. 2015;36(4):811-6. doi:10.3174/ajnr.A4173

7. Webster BS, Choi Y, Bauer AZ, et al. The cascade of medical services and associated longitudinal costs due to nonadherent magnetic resonance imaging for low back pain. *Spine (Phila Pa 1976)*. 2014;39(17):1433-40. doi:10.1097/BRS.0000000000000408

8. Webster BS, Bauer AZ, Choi Y, et al. Iatrogenic consequences of early magnetic resonance imaging in acute, work-related, disabling low back pain. *Spine (Phila Pa 1976)*. 2013;38(22):1939-46. doi:10.1097/BRS.0b013e3182a42eb6

9. McCaffery K, Nickel B, Moynihan R, et al. How different terminology for ductal carcinoma in situ impacts women's concern and treatment preferences: a randomised comparison within a national community survey. *BMJ Open*. 2015;5(11):e008094. doi:10.1136/bmjopen-2015-008094

For peer review only

10. Copp T, McCaffery K, Azizi L, et al. Influence of the disease label 'polycystic ovary syndrome' on intention to have an ultrasound and psychosocial outcomes: a randomised online study in young women. *Hum Reprod*. 2017;32(4):876-84. doi:10.1093/humrep/dex029

11. Scherer LD, Finan C, Simancek D, et al. Effect of "Pink Eye" Label on Parents' Intent to Use Antibiotics and Perceived Contagiousness. *Clin Pediatr (Phila)*. 2016;55(6):543-8. doi:10.1177/0009922815601983

12. Scherer LD, Zikmund-Fisher BJ, Fagerlin A, et al. Influence of "GERD" label on parents' decision to medicate infants. *Pediatrics*. 2013;131(5):839-45. doi:10.1542/peds.2012-3070

13. Scherer AM, Knaus M, Zikmund-Fisher BJ, et al. Effects of Influenza Strain Label on Worry and Behavioral Intentions. *Emerg Infect Dis*. 2017;23(8):1425-6. doi:10.3201/eid2308.170364

14. Azam N, Harrison M. Patients' perspectives on injuries. *Emerg Med J*. 2011;28(7):601-3. doi:10.1136/emj.2009.082032

15. Nickel B, Barratt A, Copp T, et al. Words do matter: a systematic review on how different terminology for the same condition influences management preferences. *BMJ Open*. 2017;7(7):e014129. doi:10.1136/bmjopen-2016-014129

16. Rosenkrantz AB. Differences in Perceptions Among Radiologists, Referring Physicians, and Patients Regarding Language for Incidental Findings Reporting. *AJR Am J Roentgenol*. 2017;208(1):140-3. doi:10.2214/AJR.16.16633

17. Bossen JK, Hageman MG, King JD, et al. Does rewording MRI reports improve patient understanding and emotional response to a clinical report? *Clin Orthop Relat Res*. 2013;471(11):3637-44. doi:10.1007/s11999-013-3100-x

18. Eysenbach G. Improving the quality of Web surveys: the Checklist for Reporting Results of Internet E-Surveys (CHERRIES). *Journal of medical Internet research*. 2004;6(3):e34-e. doi:10.2196/jmir.6.3.e34

19. Brinjikji W, Diehn FE, Jarvik JG, et al. MRI Findings of Disc Degeneration are More Prevalent in Adults with Low Back Pain than in Asymptomatic Controls: A Systematic Review and Meta-Analysis. *AJNR Am J Neuroradiol*. 2015;36(12):2394-9. doi:10.3174/ajnr.A4498

For peer review only

20. Jarvik JG, Comstock BA, James KT, et al. Lumbar Imaging With Reporting Of Epidemiology (LIRE)--Protocol for a pragmatic cluster randomized trial. *Contemp Clin Trials*. 2015;45(Pt B):157-63. doi:10.1016/j.cct.2015.10.003

21. Linton SJ, Boersma K. Early Identification of Patients at Risk of Developing a Persistent Back Problem: The Predictive Validity of The Örebro Musculoskeletal Pain Questionnaire. *The Clinical Journal of Pain*. 2003;19(2):80-6.

22. Darlow B, Perry M, Mathieson F, et al. The development and exploratory analysis of the Back Pain Attitudes Questionnaire (Back-PAQ). *BMJ Open*. 2014;4(5).

23. Robinson HS, Dagfinrud H. Reliability and screening ability of the StarT Back screening tool in patients with low back pain in physiotherapy practice, a cohort study. *BMC Musculoskeletal Disorders*. 2017;18:232. doi:10.1186/s12891-017-1553-x

24. Hill JC, Whitehurst DGT, Lewis M, et al. Comparison of stratified primary care management for low back pain with current best practice (STarT Back): a randomised controlled trial. *The Lancet*. 2011;378(9802):1560-71. doi:10.1016/s0140-6736(11)60937-9

25. Sullivan MJ, Bishop SR. The Pain Catastrophizing Scale: Development and Validation.

26. Bostick GP, Schopflocher D, Gross DP. Validity evidence for the back beliefs questionnaire in the general population. *Eur J Pain*. 2013;17(7):1074-81. doi:10.1002/j.1532-2149.2012.00275.x

27. Karran EL, Medalian Y, Hillier SL, et al. The impact of choosing words carefully: an online investigation into imaging reporting strategies and best practice care for low back pain. *PeerJ*. 2017;5:e4151. doi:10.7717/peerj.4151

28. Medalian Y, Moseley GL, Karran EL. An online investigation into the impact of adding epidemiological information to imaging reports for low back pain. *Scandinavian Journal of Pain*. 2019;19(3):629. doi:https://doi.org/10.1515/sjpain-2019-0023

29. Lemmers GPG, van Lankveld W, Westert GP, et al. Imaging versus no imaging for low back pain: a systematic review, measuring costs, healthcare utilization and absence from work. *Eur Spine J*. 2019. doi:10.1007/s00586-019-05918-1

For peer review only

30. Fried JG, Andrew AS, Ring NY, et al. Changes in Primary Care Health Care Utilization after Inclusion of Epidemiologic Data in Lumbar Spine MR Imaging Reports for Uncomplicated Low Back Pain. *Radiology*. 2018;287(2):563-9. doi:10.1148/radiol.2017170722

31. McCullough BJ, Johnson GR, Martin BI, et al. Lumbar MR imaging and reporting epidemiology: do epidemiologic data in reports affect clinical management? *Radiology*. 2012;262(3):941-6. doi:10.1148/radiol.11110618

32. Heidenreich PA, Gholami P, Sahay A, et al. Clinical reminders attached to echocardiography reports of patients with reduced left ventricular ejection fraction increase use of beta-blockers: a randomized trial. *Circulation*. 2007;115(22):2829-34. doi:10.1161/CIRCULATIONAHA.106.684753

33. Heidenreich PA, Chacko M, Goldstein MK, et al. ACE inhibitor reminders attached to echocardiography reports of patients with reduced left ventricular ejection fraction. *Am J Med*. 2005;118(9):1034-7. doi:10.1016/j.amjmed.2004.12.028

34. Stock JL, Wand CE, Coderre JA, et al. Clinical Reporting to Primary Care Physicians Leads to Increased Use and Understanding of Bone Densitometry and Affects the Management of Osteoporosis. *Annals of Internal Medicine*. 1998;128(12_Part_1). doi:10.7326/0003-4819-128-12_Part_1-199806150-00006

35. Ramsay CR, Eccles M, Grimshaw JM, et al. Assessing the long-term effect of educational reminder messages on primary care radiology referrals. *Clinical Radiology*. 2003;58(4):319-21.

36. Eccles M, Steen N, Grimshaw J, et al. Effect of audit and feedback, and reminder messages on primary-care radiology referrals: a randomised trial. *Lancet*. 2001;357(9266):1406-9.

37. Jarvik JG, Meier EN, James KT, et al. The Effect of Including Benchmark Prevalence Data of Common Imaging Findings in Spine Image Reports on Health Care Utilization Among Adults Undergoing Spine Imaging: A Stepped-Wedge Randomized Clinical Trial. *JAMA Network Open*. 2020;3(9):e2015713-e. doi:10.1001/jamanetworkopen.2020.15713

38. Gross DP, Ferrari R, Russell AS, et al. A population-based survey of back pain beliefs in Canada. *Spine (Phila Pa 1976)*. 2006;31(18):2142-5. doi:10.1097/01.brs.0000231771.14965.e4

39. Darlow B, Perry M, Stanley J, et al. Cross-sectional survey of attitudes and beliefs about back pain in New Zealand. *BMJ Open*. 2014;4(5):e004725. doi:10.1136/bmjopen-2013-004725

For peer review only

40. Buchbinder R, Jolley D. Effects of a media campaign on back beliefs is sustained 3 years after its cessation. *Spine (Phila Pa 1976)*. 2005;30(11):1323-30. doi:10.1097/01.brs.0000164121.77862.4b

41. Buchbinder R, Jolley D, Wyatt M. Population based intervention to change back pain beliefs and disability: three part evaluation. *BMJ*. 2001;322(7301):1516-20. doi:10.1136/bmj.322.7301.1516

42. Jenkins HJ, Hancock MJ, Maher CG, et al. Understanding patient beliefs regarding the use of imaging in the management of low back pain. *Eur J Pain*. 2016;20(4):573-80. doi:10.1002/ejp.764

43. Buchbinder R, Jolley D. Population based intervention to change back pain beliefs: three year follow up population survey. *BMJ*. 2004;328(7435):321. doi:10.1136/bmj.328.7435.321

44. Gross DP, Russell AS, Ferrari R, et al. Evaluation of a Canadian Back Pain Mass Media Campaign. *Spine*. 2010;35(8):906-13. doi:10.1097/BRS.0b013e3181c91140

45. Cabarrus M, Naeger DM, Rybkin A, et al. Patients Prefer Results From the Ordering Provider and Access to Their Radiology Reports. *J Am Coll Radiol*. 2015;12(6):556-62. doi:10.1016/j.jacr.2014.12.009

46. Johnson AJ, Easterling D, Williams LS, et al. Insight from patients for radiologists: improving our reporting systems. *J Am Coll Radiol*. 2009;6(11):786-94. doi:10.1016/j.jacr.2009.07.010

47. Johnson AJ, Frankel RM, Williams LS, et al. Patient Access to Radiology Reports: What Do Physicians Think? *J*. 2010;7(4):281-9. doi:http://dx.doi.org/10.1016/j.jacr.2009.10.011

48. Henshaw D, Okawa G, Ching K, et al. Access to radiology reports via an online patient portal: Experiences of referring physicians and patients. *J*. 2015;12(6):582-6.e1. doi:http://dx.doi.org/10.1016/j.jacr.2015.01.015

49. Zanaboni P, Ngangue P, Mbemba GIC, et al. Methods to Evaluate the Effects of Internet-Based Digital Health Interventions for Citizens: Systematic Review of Reviews. *J Med Internet Res*. 2018;20(6):e10202. doi:10.2196/10202

50. Miles RC, Hippe DS, Elmore JG, et al. Patient Access to Online Radiology Reports: Frequency and Sociodemographic Characteristics Associated with Use. *Acad Radiol*. 2016;23(9):1162-9. doi:10.1016/j.acra.2016.05.005

51. Oh SC, Cook TS, Kahn CE, Jr. PORTER: a Prototype System for Patient-Oriented Radiology Reporting. *J Digit Imaging*. 2016;29(4):450-4. doi:10.1007/s10278-016-9864-2

For peer review only - http://bmjopen.bmj.com/site/about/guidelines.xhtml

52. Darlow B, Fullen BM, Dean S, et al. The association between health care professional attitudes and beliefs and the attitudes and beliefs, clinical management, and outcomes of patients with low back pain: a systematic review. *Eur J Pain*. 2012;16(1):3-17. doi:10.1016/j.ejpain.2011.06.006

53. Rajasekaran S, Dilip Chand Raja S, Pushpa BT, et al. The catastrophization effects of an MRI report on the patient and surgeon and the benefits of 'clinical reporting': results from an RCT and blinded trials. *Eur Spine J*. 2021. doi:10.1007/s00586-021-06809-0

54. Nakashima H, Yukawa Y, Suda K, et al. Abnormal findings on magnetic resonance images of the cervical spines in 1211 asymptomatic subjects. *Spine (Phila Pa 1976)*. 2015;40(6):392-8. doi:10.1097/BRS.0000000000000775

55. Culvenor AG, Oiestad BE, Hart HF, et al. Prevalence of knee osteoarthritis features on magnetic resonance imaging in asymptomatic uninjured adults: a systematic review and meta-analysis. *Br J Sports Med*. 2018. doi:10.1136/bjsports-2018-099257

56. Girish G, Lobo LG, Jacobson JA, et al. Ultrasound of the shoulder: asymptomatic findings in men. *AJR Am J Roentgenol*. 2011;197(4):W713-9. doi:10.2214/AJR.11.6971

For peer review only

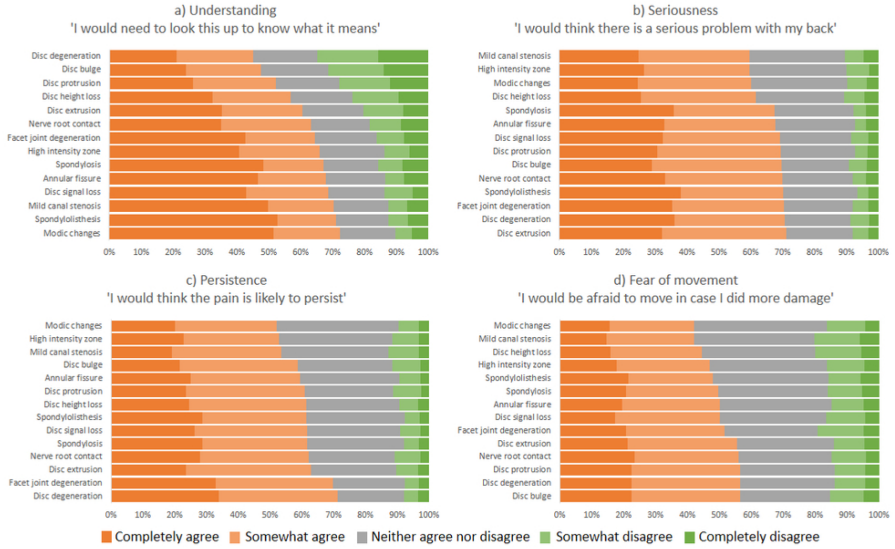

Figure 1: Responses to questions regarding level of understanding, seriousness, persistence of pain and fear of movement for the 14 radiology terms

Note: For each term, we asked four questions with a common opening ('If this term was in my report I would...') to evaluate a) understanding ('....need to look this term up to know what it means'); b) worry about seriousness ('...be worried there is a serious problem with my back'); c) worry about persistence ('...be worried my pain would persist') and d) fear of movement ('...be afraid to move in case I did more damage').

46x26mm (600 x 600 DPI)

Cherries checklist – Consumer understanding of terms used in imaging reports requested for low back pain

	Category	Checklist item	Page(s)
1	Design	**Describe survey design** Describe target population, sample frame. Is the sample a convenience sample? (In 'open' surveys this is most likely).	6-11
2	IRB (Institutional Review Board) approval and informed consent process	**IRB approval** Mention whether the study has been approved by an IRB.	10
3		**Informed consent** Describe the informed consent process. Where were the participants told the length of time of the survey, which data were stored and where and for how long, who the investigator was, and the purpose of the study?	10
4		**Data protection** If any personal information was collected or stored, describe what mechanisms were used to protect unauthorised access.	N/A
5	Development and pre-testing	**Development and testing** Stated how the survey was developed, including whether the usability and technical functionality of the electronic questionnaire had been tested before fielding the questionnaire	7
6	Recruitment process and description of the sample having access to the questionnaire	**Open survey vs closed survey** An 'open survey' is a survey open for each visitor of a site, while a closed survey is only open to a sample which the investigator knows (password-protected survey).	6-7
7		**Contact mode** Indicate whether or not the initial contact with the potential participants was made on the internet (Investigators may also send out questionnaires by mail and allow for Web-based data entry).	6-7
8		**Advertising the survey** How/where was the survey announced or advertised? Some examples are offline media (newspapers), or online (mailing lists – if yes, which ones?) or banner ads (where were these banner ads posted and what did they look like?). It is important to know the wording of the announcement as it will heavily influence who chooses to participate. Ideally the survey announcement should be published as an appendix.	6-7
9	Survey administration	**Web/email** State the type of e-survey (eg one posted on a website, or one sent out through email). If it is an email survey, were the responses entered manually into a database, or was there an automatic method for capturing responses?	6
10		**Context** Describe the website (for mailing list/newsgroup) in which the survey was posted. What is the website about, who is visiting it, what are visitors normally looking for? Discuss to what degree the content of the website could pre-select	N/A

For peer review only

		the sample or influence the result. For example, a survey about vaccination on an anti-immunisation website will have different results from a web survey conducted on a N/A government website.	
11		**Mandatory/voluntary**<br>Was it a mandatory survey to be filled in by every visitor who wanted to enter the website, or was it a voluntary survey?	N/A
12		**Incentives**<br>Were any incentives offered (eg monetary, prizes, or non-monetary incentives such as an offer to provide the survey results)?	6-7
13		**Time/Date**<br>In what timeframe were the data collected?	6
14		**Randomisation of items or questionnaires**<br>To prevent biases items can be randomised or alternated	7
15		**Adaptive questioning**<br>Use adaptive questioning (certain items, or only conditionally displayed based on responses to other items) to reduce number and complexity of the questions	6-7
16		**Number of items**<br>What was the number of questionnaire items per page? The number of items is an important factor for the completion rate.	7-8
17		**Number of screens (pages)**<br>Over how many pages was the questionnaire distributed? The number of items is an important factor for the completion rate.	N/A
18		**Completeness check**<br>It is technically possible to do consistency or completeness checks before the questionnaire is submitted. Was this done, and if 'yes' how (usually JAVAscript)? An alternative is to check for completeness after the questionnaire has been submitted (and highlight mandatory items). If this has been done, it should be reported. All items should provide a non-response option such as 'not applicable' or 'rather not say' and selection of one response option should be enforced.	9
19		**Review step**<br>State whether respondents were able to review and change their answers (eg through a Back button or a Review step which displays a summary of the responses and asks the respondents if they are correct).	10
20	Response rates	**Unique site visitor**<br>If you provide view rates or participation rates, you need to define how you determined a unique visitor. There are different techniques available, based on IP addresses or cookies or both.	N/A

For peer review only

21		**View rate**<br>**(ratio of unique survey visitors/unique site visitors)**<br>Requires counting unique visitors to the first page of the survey, divided by the number of unique site visitors (not page views!). It is not unusual to have view rates of less than 0.1% if the survey is voluntary.	N/A
22		**Participation rate**<br>**(Ratio of unique visitors who agreed to participate/unique first survey page visitors)**<br>Count the unique number of people who filled in the first survey page (or agreed to participate, for example by checking a checkbox), divided by visitors who visit the first page of the survey (or the informed consents page, if present). This can also be called 'recruitment' rate.	N/A
23		**Completion rate**<br>**(Ratio of users who finished the survey/users who agreed to participate)**<br>The number of people submitting the last questionnaire page, divided by the number of people who agreed to participate (or submitted the first survey page). This is only relevant if there is a separate 'informed consent' page or if the survey goes over several pages. This is a measure for attrition. Note that 'completion' can involve leaving questionnaire items blank. This is not a measure for how completely questionnaires were filled in. (If you need a measure for this use the word 'completeness rate'.)	11
24	Preventing multiple entries from the same individual	**Cookies used**<br>Indicate whether cookies were used to assign a unique user identifier to each client computer. If so, mention the page on which the cookie was set and read, and how long the cookie was valid. Were duplicate entries avoided by preventing users access to the survey twice; or were duplicate database entries having the same user ID eliminated before analysis? If the latter, which entries were kept for analysis (eg the first entry or the most recent)?	N/A
25		**IP check**<br>Indicate whether the IP address of the client computer was used to identify potential duplicate entries from the same user. If so, mention the period of time for which no two entries from the same IP address were allowed (eg 24 hours). Were duplicate entries avoided by preventing users with the same IP address access to the survey twice; or were duplicate database entries having the same IP address within a given period of time eliminated before analysis? If the latter, which entries were kept for analysis (eg the first entry or the most recent)?	9
26		**Log file analysis**<br>Indicate whether other techniques to analyse the log file for identification of multiple entries were used. If so, please describe.	N/A
27		**Registration**<br>In 'closed' (non-open) surveys, users need to login first and it is easier to prevent duplicate entries from the same user. Describe how this was done. For example, was the survey never displayed a second time once the user had filled	N/A

For peer review only

		it in, or was the username stored together with the survey results and later eliminated? If the latter, which entries were kept for analysis (eg the first entry or the most recent)?	
28	Analysis	**Handling of incomplete questionnaires**<br>Were only completed questionnaires analysed? Were questionnaires which terminated early (where, for example, users did not go through all questionnaire pages) also analysed?	9, 11
29		**Questionnaires submitted with an atypical timestamp**<br>Some investigators may measure the time people needed to fill in a questionnaire and exclude questionnaires that were submitted too soon. Specify the timeframe that was used as a cut-off point and describe how this point was determined.	N/A
30		**Statistical correction**<br>Indicate whether any methods such as weighting of items or propensity scores have been used to adjust for the non-representative sample; if so, please describe the methods.	N/A

For peer review only

BMJ Open

Consumer understanding of terms used in imaging reports requested for low back pain: a cross-sectional survey

Journal:	*BMJ Open*
Manuscript ID	bmjopen-2021-049938.R2
Article Type:	Original research
Date Submitted by the Author:	13-Aug-2021
Complete List of Authors:	Farmer, Caitlin; Monash University, Department of Epidemiology and Preventive Medicine, School of Public Health and Preventive Medicine; Monash University, Monash-Cabrini Department of Musculoskeletal Health and Clinical Epidemiology, Cabrini Health<br>O'Connor, Denise; Monash University, Department of Epidemiology and Preventive Medicine, School of Public Health and Preventive Medicine; Monash University, Monash-Cabrini Department of Musculoskeletal Health and Clinical Epidemiology, Cabrini Health<br>Lee, Hopin; University of Oxford, Centre for Statistics in Medicine, Rehabilitation Research in Oxford, Nuffield Department of Orthopaedics Rheumatology and Musculoskeletal Sciences (NDORMS), ; The University of Newcastle, School of Medicine and Public Health<br>McCaffery, Kirsten; The University of Sydney, Sydney Health Literacy Lab, School of Public Health<br>Maher, Christopher; The University of Sydney, School of Public Health; The University of Sydney Institute for Musculoskeletal Health<br>Newell, Dave; AECC University College<br>Cashin, Aidan; Neuroscience Research Australia, Centre for Pain IMPACT; University of New South Wales, Prince of Wales Clinical School<br>Byfield, David; University of South Wales Faculty of Life Sciences and Education<br>Jarvik, Jeffrey; University of Washington, Departments of Radiology, Neurological Surgery and Health Services, School of Medicine; University of Washington, UW Clinical Learning, Evidence And Research (CLEAR) Center for Musculoskeletal Disorders<br>Buchbinder, Rachelle; Monash University Faculty of Medicine Nursing and Health Sciences, Dept of Epidemiology and Preventive Medicine, School of Public Health and Preventive Medicine; Monash University, Monash-Cabrini Department of Musculoskeletal Health and Clinical Epidemiology, Cabrini Health
**Primary Subject Heading**:	Radiology and imaging
Secondary Subject Heading:	Public health
Keywords:	Diagnostic radiology < RADIOLOGY & IMAGING, Back pain < ORTHOPAEDIC & TRAUMA SURGERY, Musculoskeletal disorders < ORTHOPAEDIC & TRAUMA SURGERY

SCHOLARONE™
Manuscripts

I, the Submitting Author has the right to grant and does grant on behalf of all authors of the Work (as defined in the below author licence), an exclusive licence and/or a non-exclusive licence for contributions from authors who are: i) UK Crown employees; ii) where BMJ has agreed a CC-BY licence shall apply, and/or iii) in accordance with the terms applicable for US Federal Government officers or employees acting as part of their official duties; on a worldwide, perpetual, irrevocable, royalty-free basis to BMJ Publishing Group Ltd ("BMJ") its licensees and where the relevant Journal is co-owned by BMJ to the co-owners of the Journal, to publish the Work in this journal and any other BMJ products and to exploit all rights, as set out in our licence.

The Submitting Author accepts and understands that any supply made under these terms is made by BMJ to the Submitting Author unless you are acting as an employee on behalf of your employer or a postgraduate student of an affiliated institution which is paying any applicable article publishing charge ("APC") for Open Access articles. Where the Submitting Author wishes to make the Work available on an Open Access basis (and intends to pay the relevant APC), the terms of reuse of such Open Access shall be governed by a Creative Commons licence – details of these licences and which Creative Commons licence will apply to this Work are set out in our licence referred to above.

Other than as permitted in any relevant BMJ Author's Self Archiving Policies, I confirm this Work has not been accepted for publication elsewhere, is not being considered for publication elsewhere and does not duplicate material already published. I confirm all authors consent to publication of this Work and authorise the granting of this licence.

For peer review only

Consumer understanding of terms used in imaging reports requested for low back pain: a cross-sectional survey

Caitlin Farmer[1,2], Denise O'Connor[1,2], Hopin Lee[3,4], Kirsten McCaffery[5], Christopher Maher[6,7], Dave Newell[8], Aidan Cashin[9,10], David Byfield[11], Jeffrey G. Jarvik[12,13], Rachelle Buchbinder[1,2]

[1]Department of Epidemiology and Preventive Medicine, School of Public Health and Preventive Medicine, Monash University, Melbourne, Australia.

[2]Monash Department of Clinical Epidemiology, Cabrini Health, Melbourne, Australia.

[3]Centre for Statistics in Medicine, Rehabilitation Research in Oxford, Nuffield Department of Orthopaedics Rheumatology and Musculoskeletal Sciences (NDORMS), University of Oxford, Oxford, United Kingdom.

[4]School of Medicine and Public Health, University of Newcastle, Newcastle, Australia.

[5]Sydney Health Literacy Lab, Wiser Healthcare, Sydney School of Public Health, Faculty of Medicine and Health, University of Sydney, Sydney, Australia

[6]School of Public Health, University of Sydney, Sydney, Australia.

[7]Institute for Musculoskeletal Health, Sydney, Australia.

[8]AECC University College, Dorset, United Kingdom.

[9]Centre for Pain IMPACT, Neuroscience Research Australia, Sydney, Australia.

[10]Prince of Wales Clinical School, Faculty of Medicine, University of New South Wales, Sydney, Australia.

[11]Faculty of Life Sciences and Education University of South Wales, Treforest, United Kingdom.

[12]Departments of Radiology, Neurological Surgery and Health Services, School of Medicine, University of Washington, United States.

[13]UW Clinical Learning, Evidence And Research (CLEAR) Center for Musculoskeletal Disorders, Seattle, WA, United States.

Corresponding author:

Caitlin Farmer

Monash Department of Clinical Epidemiology

4 Drysdale St

Malvern VIC 3144

Australia

caitlin.farmer1@monash.edu

Word Count: 4,103 excluding title page, abstract, tables and references

For peer review only - http://bmjopen.bmj.com/site/about/guidelines.xhtml

ABSTRACT

Objectives: To investigate 1) self-reported societal comprehension of common and usually non-serious terms found in lumbar spine imaging reports and 2) its relationship to perceived seriousness, likely persistence of low back pain (LBP), fear of movement, back beliefs and history and intensity of low back pain.

Design: Cross-sectional online survey of the general public.

Setting: Five English-speaking countries: United Kingdom, United States, Canada, New Zealand, Australia.

Participants: Adults (age > 18 years) with or without a history of LBP recruited in April 2019 with quotas for country, age and gender.

Primary and secondary outcome measures: Self-reported understanding of 14 terms (annular fissure, disc bulge, disc degeneration, disc extrusion, disc height loss, disc protrusion, disc signal loss, facet joint degeneration, high intensity zone, mild canal stenosis, modic changes, nerve root contact, spondylolisthesis and spondylosis) commonly found in lumbar spine imaging reports. For each term we also elicited worry about its seriousness, and whether its presence would indicate pain persistence and prompt fear of movement.

Results: From 774 responses we included 677 (87.5%) with complete and valid responses. 577 (85%) participants had a current or past history of LBP of whom 251 (44%) had received lumbar spine imaging. Self-reported understanding of all terms was poor. At best, 235 (35%) reported understanding the term ‘disc degeneration’, while only 71 (10.5%) reported understanding the term ‘modic changes’. For all terms, a moderate to large proportion of participants (range 59-71%), considered they indicated a serious back problem, that pain might persist (range 52-71%) and they would be fearful of movement (range 42-57%).

Conclusion: Common and usually non-serious terms in lumbar spine imaging reports are poorly understood by the general population and may contribute to the burden of LBP.

Trial registration: This study was prospectively registered with the Australian & New Zealand Clinical Trials Registry (12619000545167).

STRENGTHS AND LIMITATIONS OF THIS STUDY

- This is the first study to directly measure societal self-reported understanding of 14 commonly used terms found in lumbar spine imaging reports and its relationship to worry regarding the seriousness and persistence of low back pain and being fearful of movement.
- Participants were a large representative sample from five high-income English-speaking countries with quotas for age and gender suggesting our results are likely generalisable to those settings but may or may not be generalisable to other settings.
- Self-reported understanding may not truly reflect a person's understanding of the terms, and the use of a survey sampling company may have introduced some bias.
- Although all terms that we investigated are findings that commonly exist in the asymptomatic population, some are more common in people with low back pain, and this may have influenced participants' responses.

For peer review only - http://bmjopen.bmj.com/site/about/guidelines.xhtml

INTRODUCTION

Low back pain (LBP) is a common, costly problem and a significant contributor to global disability.(1) Most LBP is labelled 'non-specific', reflecting an inability to identify a specific cause in most cases.(2) In the absence of features suggestive of a serious cause, clinical practice guidelines consistently recommend against any form of lumbar spine imaging as it is unlikely to provide meaningful information about the cause of pain or guide treatment.(3-5) Imaging often reveals age-related changes such as disc degeneration, disc bulge and facet joint degeneration that are also common in asymptomatic people.(6)

Lumbar spine imaging also carries risk of iatrogenic harm. This includes not only exposure to ionising radiation with plain radiographs and CT scans, but the way findings are described may lead to clinician and patient anxiety about perceived seriousness, prompt further unnecessary tests, unwarranted diagnostic labels and unnecessary treatment.(7, 8)

While few published studies have investigated consumer understanding and views regarding terminology in lumbar spine imaging findings in people with LBP, several studies have investigated these issues for other conditions.(9-14) A systematic review investigating how different terms for various conditions influence management preferences found that use of more medicalised or precise terms generally results in greater anxiety and perceived severity, and preferences towards more invasive care.(15) Ambiguous terminology for incidental low-risk liver lesions reported in abdominal CT reports was found to increase patient and referrer anxiety and a greater likelihood of follow-up testing.(16) In contrast, simplified upper limb MRI reports using more understandable and less 'emotive' language (e.g., 'expected age-related changes' rather than 'hypertrophic degenerative changes') have been found to reduce worry, improve comprehension and help patients feel more in control of their health.(17)

For peer review only

As patients are increasingly accessing their imaging reports, it is important to understand how commonly reported terms are interpreted by patients. The primary aim of this study was to investigate self-reported understanding of terms that are both commonly found in lumbar spine imaging reports and are usually of little clinical relevance. We also investigated the relationship between self-reported understanding of these terms and their perceived seriousness, perceptions about the likelihood of pain persistence and whether they would result in being fearful of movement. Our secondary aim was to describe the association between self-reported understanding of the terms and back beliefs, and history and intensity of LBP. We also elicited views about access to imaging reports and whether they should be understandable to patients and include context.

METHODS

Design

This study was a cross-sectional online survey of a random sample of the general public in five English-speaking countries: Australia, New Zealand, the United States, United Kingdom, Canada. It was conducted and reported in accordance with the Checklist for Reporting Results of Internet E-Surveys (CHERRIES) guideline (18).

Participants and recruitment

Participants were recruited in April 2019 via an online survey sampling company (Dynata). Dynata is a data company that maintains a research and marketing database. It recruits participants via email based on the requirements of the research involved. Upon survey completion, Dynata provides participant incentives that allow redemption of items such as gift cards and contributions to charity. English-speaking adults aged 18 and over with or without a

For peer review only

For peer review only - http://bmjopen.bmj.com/site/about/guidelines.xhtml

current or past history of back pain were recruited via email with quotas established for country, age and gender to ensure a representative sample.

The survey was conducted in Qualtrics and no identifying information was provided to the research team.

Survey instrument

Pilot testing conducted in a convenience sample of ten members of the general public resulted in minor changes. The survey included nine demographic questions (age in years, gender (female/male/other/prefer not to say), education level (finished high school/technical/trade certificate or diploma, university (Bachelor), post-graduate university qualifications), employment status (full time/part time/unemployed/student/home duties/retired (not due to ill health)/retired (due to ill health)/other), health care provider for people with LBP (yes/no), LBP history (current, past, never) and for those with a history of LBP we asked about average pain intensity (measured on a 0 to 100 point VAS where higher scores indicate greater pain), LBP imaging (Yes/No/Don't Know) and treatment (surgery and injections).

Four statements were presented for each of 14 radiology terms (annular fissure, disc bulge, disc degeneration, disc extrusion, disc height loss, disc protrusion, disc signal loss, facet joint degeneration, high intensity zone, mild canal stenosis, modic changes, nerve root contact, spondylolisthesis and spondylosis). These terms were chosen on the basis that they are commonly found in lumbar imaging reports of people with LBP but are also common findings in asymptomatic populations and are usually considered benign or of unclear clinical significance.(6, 19, 20) For each question, order of presentation of the terms was randomised to reduce order effect bias.

For peer review only - http://bmjopen.bmj.com/site/about/guidelines.xhtml

For each item, we elicited the participant's level of agreement on a 5-point Likert scale (1=Completely disagree, 2=Somewhat disagree, 3=Neither agree nor disagree, 4=Somewhat agree, 5=Completely agree) to the following four statements:

1. I would need to look this term up to know what it means (Understanding)
2. I would be worried there is a serious problem with my back (Seriousness)
3. I would be worried my pain isn't going to get any better (Persistence)
4. I would be afraid to move my back in case I did more damage (Fear of movement)

Items 2 and 3 were derived from questionnaires for musculoskeletal conditions or LBP that assess perceived seriousness or likely persistence of pain.(21-23) For example the third item is similar to items in the STarT Back Tool ('I feel my back pain is terrible and it's never going to get any better'),(24) Pain Catastrophizing Scale ('It's terrible and I think it's never going to get any better')(25) and the Back-PAQ ('There is a high chance that an episode of back pain will not resolve').(22)

We also elicited opinions (yes/no/don't know) about whether people should have access to their imaging reports; whether imaging reports should be written so they can be understood by lay people; and whether including epidemiological information about common findings in people without back pain would be helpful in interpreting results.

The Back Beliefs Questionnaire (BBQ) is a 14 item tool that measures beliefs about back pain.(26) Responses to each item are measured on a 5-point Likert agreement scale (completely disagree to completely agree). The BBQ is scored from nine of the 14 items with higher scores indicating more positive beliefs (score range 9 to 45).

For peer review only

Sample size and analysis

A minimum sample size of 590 was required to detect 70% ± 10% prevalence of not understanding a term. Assuming 10% drop out or non-completion of surveys, our target was 650 participants. This sample size also provided enough power to investigate associations between variables.

Raw data files were reviewed for evidence of duplicate answers including duplicate internet protocol (IP) addresses prior to inclusion in the analysis. Descriptive statistics were used to summarise the participant characteristics, responses to statements about radiological terms, back beliefs and opinions regarding imaging reports. Data were excluded for any participant who provided the same answer for every item of the BBQ as this type of response is invalid and likely indicates that the questionnaire responses for other items were also invalid.

Responses of either completely disagree or somewhat disagree with the statement 'I would need to look this up to know what it means' were considered to indicate self-reported understanding of a term.

Responses for each item were dichotomised with ‘completely disagree’ and ‘somewhat disagree’ coded as zero and ‘neither agree nor disagree’/’somewhat agree’/’completely agree’ coded as one. For self-reported understanding, we combined a neutral response (neither agree nor disagree) with somewhat or completely agree that the person would need to look the term up to know what it means as we considered a neutral response more likely indicated that a respondent didn’t understand a term. For items regarding worry about seriousness, persistence of pain and fear of movement, we combined a neutral response with somewhat or completely

For peer review only

For peer review only - http://bmjopen.bmj.com/site/about/guidelines.xhtml

agree with these statements (ie, worried about seriousness, pain persistence or being fearful of movement).

The total number and percent of positive responses for all 14 radiology terms were calculated for each participant. For the item about understanding, a score of 0 (0%) indicates that respondents reported that they would not need to look up any of the 14 terms (high level of understanding), while a score of 14 (100%) indicates they would need to look up all terms (low understanding). For items regarding worry about seriousness, persistence of pain and fear of movement, a score of 0 indicates that none of the terms elicited worry about seriousness, persistence of pain and/or movement, while a score of 14 indicates that all terms elicited concerns about seriousness, persistence of pain and/or movement.

Spearman's rank correlation was used to investigate the correlation between responses to the four statements regarding the terms. Multivariate regression analysis was used to investigate the association between self-reported understanding of terms and perceived seriousness, risk of persistence and fear of movement (measured by proportion of positive responses), and their association with participant demographics, history of LBP and BBQ score.

Sensitivity analysis

To ensure that the dichotomisation was an appropriate method of handling the data, in particular the neutral responses, we performed two post-hoc sensitivity analyses following comments from the reviewers of the manuscript. In the first, for the questions regarding seriousness, pain persistence and fear of movement, we combined the neutral responses with the strongly disagree and somewhat disagree responses and compared them to the somewhat agree and strongly agree responses. In the second sensitivity analysis we removed neutral

For peer review only

responses altogether and compared the answers of strongly disagree and somewhat disagree with somewhat agree and strongly agree.

Ethics

This project was approved by the Monash University Human Research Ethics Committee (ID 13896). Survey completion was considered consent to participate and participants were able to review their answers prior to submission.

Patient and public involvement

Consumer feedback about questionnaire burden and time requirements was incorporated into survey design. Survey participants were advised how to access results of the study in the accompanying explanatory statement.

RESULTS

Of 774 responses, we excluded 97 (12.5%) due to incomplete data (n=35, 4.5%), invalid BBQ responses (n=59, 7.6%) and age given as under 18 years (n=3, 0.4%). No duplicate responses were identified. We were unable to calculate a response rate as Dynata were unable to provide precise data about how often the survey was available to users via either email or their dashboard.

Responses from 677 participants (52% female) were analysed (Table 1). There were no significant between-country differences regarding demographic characteristics. 577 participants (85%) reported past or current LBP, and 279 (41%) reported having LBP at the time of survey completion. The mean (SD) BBQ score for the whole cohort was 25.0 (6.4). Pain intensity was higher in those who reported current compared with past LBP (mean (SD):

For peer review only

54.7 (21.3), n=279, versus 44.2 (22.1), n=298, respectively, p<0.001). Of those with a history of LBP, 251 (44%) reported receipt of lumbar spine imaging, 81 (14%) reported receipt of spine injection/s and 27 (5%) reported undergoing spine surgery.

Participant views regarding terms

Most participants (n=598, 88%) somewhat or completely agreed they would need to look up at least one of the terms to know what it means, while only 3% (n=17) somewhat or completely disagreed with this statement for all terms (Figure 1). Overall, 41 (6%) participants provided neutral answers for all comprehension questions (i.e., neither agree nor disagree).

The proportion who reported understanding the meaning of terms ranged from 35% (n=235) for the term 'disc degeneration' to 10.5% (n=71) for 'modic changes' (Figure 1a). The same proportion (88%) somewhat or completely agreed they would be worried about a serious problem with their back for at least one term and only 3% (n=17) disagreed with this statement for all terms (Figure 1b). Three terms, 'disc extrusion', 'disc degeneration' and 'facet joint degeneration', elucidated the most worry about seriousness (71% of participants). Worry regarding seriousness varied only a small amount between terms, with the number of participants disagreeing with the statement varying between only 7% and 11% for all terms.

For peer review only

Table 1: Participant demographics and back pain history and beliefs by country and overall

		USA (n=139)	Australia (n=138)	NZ (n=135)	UK (n=132)	Canada (n=133)	TOTAL (n=677)
		Mean (SD)	***Mean (SD)***	***Mean (SD)***	***Mean (SD)***	***Mean (SD)***	***Mean (SD)***
Age, years		45.4 (16.0)	45.0 (17.0)	45.9 (17.6)	47.6 (17.0)	45.4 (15.5)	45.8 (16.4)
Back pain intensity most recent episode* (0-100 scale, higher score indicates greater pain)	Current LBP	49.7 (22.4)	58.2 (20.2)	55.0 (21.7)	58.1 (22.0)	51.9 (20.2)	54.7 (21.3)
	Previous LBP	40.0 (19.7)	48.4 (22.6)	44.2 (21.3)	45.4 (24.4)	44.0 (22.6)	44.2 (22.1)
BBQ score (9-45 scale, higher score indicates better back beliefs)		25.7 (6.4)	24.3 (6.5)	25.8 (6.6)	24.6 (6.3)	24.4 (6.1)	25.0 (6.4)
		N (%)	***N (%)***	***N (%)***	***N (%)***	***N (%)***	***N (%)***
Female		75 (54)	73 (53)	70 (52)	67 (51)	69 (52)	354 (52)
University education		69 (50)	51 (37)	63 (47)	59 (45)	57 (43)	299 (44)
Employed full or part time		71 (51)	80 (58)	77 (57)	73 (55)	80 (60)	381 (56)
Worked as healthcare professional[#]		10 (7)	4 (3)	4 (3)	5 (4)	4 (3)	27 (4)
Ever had low back pain		116 (83)	119 (86)	114 (84)	113 (86)	115 (86)	577 (85)
Current low back pain		54 (38)	68 (49)	45 (33)	52 (39)	60 (45)	279 (41)
Had lumbar spine imaging*		60 (52)	60 (50)	40 (35)	38 (34)	53 (46)	251 (44)
Had lumbar spine injection*		17 (15)	18 (15)	16 (14)	21 (19)	9 (8)	81 (14)
Had lumbar spine surgery*		6 (5)	4 (3)	7 (6)	6 (5)	4 (4)	27 (5)

*Only for participants who responded 'yes' to current or previous history of low back pain

[#]Health professionals included 9 nurses, 4 doctors (1 GP), 3 health care assistants, 2 pharmacists, an occupational therapist, pharmacy technician, physical therapist, physician assistant, prescription manager, psychologist, respiratory therapist and a 'therapist'.

Abbreviations: USA – United States of America, NZ – New Zealand, UK – United Kingdom, SD – Standard Deviation, BBQ – Back beliefs questionnaire, LBP – Low back pain

For peer review only - http://bmjopen.bmj.com/site/about/guidelines.xhtml

For peer review only

For all terms, over half the participants were worried that back pain would persist if the imaging finding was present (Figure 1c). The least number of participants (51%) reported worry regarding persistence for the term ‘modic changes’, while ‘disc degeneration’ was the term which caused worry in the greatest percent of participants (71%). In total 84% of participants agreed they would be worried regarding persistence for at least one term, and 4% of participants disagreed with the statement for all terms.

For many imaging findings, participants also frequently reported they would be afraid to move their back. 74% agreed with this statement for at least one term and only 8% disagreed with this statement for all terms (Figure 1d). The greatest number of participants agreed with this statement for the term ‘disc bulge’ (57%), and 56% of participants also agreed with this statement for the terms ‘disc degeneration’, ‘disc protrusion’, ‘nerve root contact’ and ‘disc extrusion’. ‘Modic changes’ and ‘mild canal stenosis’ were the terms associated with least worry regarding movement (42% agreed with the statement).

Associations between self-reported understanding of terms and perceived seriousness, risk of pain persistence and fear of movement

There was moderate correlation between understanding of terms and worry about seriousness, pain persistence and fear of movement (Spearman’s rho 0.48, 0.39, and 0.38, respectively all $p<0.001$) (Table 2). Strong associations were present between the responses regarding worry about seriousness and pain persistence (Spearman’s rho 0.63, $p<0.001$) and pain persistence and fear of movement (Spearman’s rho 0.51, $p<0.001$) and there was a moderate association between the responses regarding worry about seriousness and fear of movement (Spearman’s rho 0.42, $p<0.001$).

For peer review only

Table 2: Association between the self-reported understanding of the 14 terms overall and perceived seriousness, pain persistence and fear of movement, Spearman's rho correlation*

	Understanding	Seriousness	Pain persistence	Fear of movement
**Understanding**	1			
**Seriousness**	0.48	1		
**Persistence**	0.39	0.63	1	
**Fear of movement**	0.38	0.42	0.51	1

**All p values <0.001.*
Values between 0.10 and 0.29 represent a weak association, 0.30-0.49 moderate and 0.5 and above a strong association.

For peer review only

Associations with demographic features, history of back pain and back beliefs

Results of the regression analysis are presented in Table 3. Higher education level was associated with greater self-reported understanding of terms, and less concern about seriousness, pain persistence and being fearful of movement. Participants with current LBP had better understanding and less fear of movement compared with those without a history of LBP. More positive back beliefs (higher BBQ score) were associated with greater self-reported understanding of terms and less worry regarding seriousness, persistence and movement. Older age was associated with greater worry about seriousness and pain persistence but less fear of movement. Male gender was associated with less worry about seriousness and movement compared with female gender.

For peer review only - http://bmjopen.bmj.com/site/about/guidelines.xhtml

Table 3: Association between self-reported understanding of the 14 terms, perceived seriousness, pain persistence and fear of movement and demographic details, history of back pain and back beliefs

	Understanding	Seriousness	Pain persistence	Fear of movement
	Regression coefficient (95% Confidence Interval)			
Education (REF - High school)				
Trade/Diploma	0.36 (-0.01, 0.73)	-0.23 (-0.40, 0.86)	**-0.70 (-1.28, -0.12)***	-0.44 (-0.94, 0.07)
Bachelor	**0.40 (0.07, 0.72)***	- 0.31 (-0.19, 0.81)	-0.37 (-0.85, 0.11)	**-0.50 (-0.92, -0.07)***
Postgraduate qualifications	**0.66 (0.23, 1.08)****	**-0.76 (-1.38, -0.14)***	**-1.23 (-1.82, -0.65)*****	**-0.94 (-1.46, -0.42)*****
LBP History (REF = Never)				
Previous LBP	0.27 (-0.21, 0.75)	0.31 (-0.34, 0.96)	0.07 (-0.56, 0.69)	-0.30 (-0.84, 0.25)
Current LBP	**0.50 (0.02, 0.97)***	0.10 (-0.55, 0.75)	-0.15 (-0.79, 0.48)	**-0.59 (-1.14, -0.04)***
BBQ score	**0.02 (0.0003, 0.04)***	**-0.04 (-0.07, -0.01)***	**-0.08 (-0.11, -0.04)*****	**-0.07 (-0.10, -0.05)*****
Age, years	0.002 (-0.007, 0.01)	**0.02 (0.01, 0.04)****	**0.03 (0.02, 0.04)*****	-0.004 (-0.01, 0.01)
Gender (REF = Female)				
Male	0.07 (-0.20, 0.33)	-0.40 (-0.84, 0.04)	-0.23 (-0.63, 0.18)	-0.17 (-0.52, 0.17)

Note: Regression estimates were obtained using the Generalised Linear Model with binomial distribution, log link and robust estimates. Bold text indicates statistically significant results, * $p < 0.05$, ** $p < 0.01$, *** $p < 0.001$. Results rounded to two decimal points or first meaningful decimal point. REF = Reference category, LBP = Low Back Pain, BBQ = Back Beliefs Questionnaire

Continuous variables (BBQ score and age): For understanding, a positive regression coefficient indicates greater self-reported understanding is associated with better back beliefs (higher BBQ score) and increased age. For seriousness, pain persistence and fear of movement, a positive regression coefficient indicates greater worry/concern is associated with poorer back beliefs (lower BBQ score) and increased age.

Categorical variables: For understanding, a positive regression coefficient indicates increased self-reported understanding compared with the reference category. For seriousness, pain persistence and fear of movement, a positive regression coefficient indicates greater worry/concern compared with the reference variable.

For peer review only - http://bmjopen.bmj.com/site/about/guidelines.xhtml

Sensitivity analysis

The sensitivity analyses (Supplementary Tables 1-4) are shown in the Supplementary file. There was still an association between understanding of terms and worry about seriousness, pain persistence and fear of movement when we combined neutral responses with somewhat and strongly disagree with the four statements, but the correlation was weak rather than moderate (Spearman's rho 0.29, 0.21 and 0.19 respectively, all $p<0.001$) (Supplementary Table 1). Strong associations remained between worry about seriousness and pain persistence (Spearman's rho 0.72, $p<0.001$) and pain persistence and fear of movement (0.60, $p<0.001$), and there was also a strong association between worry about seriousness and fear of movement (Spearman's rho 0.53, $p<0.001$).

Combining neutral responses with somewhat disagree and strongly disagree found that higher education level was again associated with greater self-reported understanding of terms but the relationship between education and seriousness, persistence and fear of movement, and the relationship of age to seriousness and persistence was no longer significant, suggesting these results should be interpreted with caution (Supplementary Table 2). However, more positive back beliefs (higher BBQ score) were again associated with greater self-reported understanding of terms and less worry regarding seriousness, pain persistence and movement. In contrast to the primary analysis, the relationship between male gender became significant for seriousness and pain persistence, suggesting the relationship between gender and these questions may also be complex.

Removal of the neutral responses from the analysis altogether led to results that were in close keeping with *a-priori* primary analysis. The only differences were that the association between understanding and seriousness and between seriousness and fear of movement

For peer review only - http://bmjopen.bmj.com/site/about/guidelines.xhtml

became strong rather than moderate (Spearman's rho 0.60 and 0.53 respectively, both $p<0.001$), the association between increasing age became significant for both seriousness and pain persistence, and male gender became significant for seriousness (Supplementary Tables 3 and 4).

Participants views about imaging reports

The majority of participants (n=605, 89%) agreed that people with LBP should have access to their radiology report, that it should be written in a way to facilitate lay understanding (n=586, 87%) and it would be useful to include information about the prevalence of common imaging findings in people without back pain (n=587, 87%).

Discussion

Our study found evidence of poor self-reported understanding of terms commonly used to describe usually benign findings reported in lumbar spine imaging reports. Most participants had concerns about their seriousness and associated the terms with persistence of pain and/or need to avoid (or be fearful of) movement. Higher education, more positive back beliefs and current LBP were all associated with greater self-reported understanding of the terms, while people with more positive back beliefs were also less concerned about seriousness, pain persistence and being fearful of movement. These results were robust to either combining neutral responses with disagreement with the statements or removing them from the analyses altogether.

Our findings, consistent with other literature that has found that language matters, (15, 16) provide support for changing how these findings are reported. Recent online scenario-based randomised experiments that provided imaging reports for 'virtual patients' with LBP found

For peer review only - http://bmjopen.bmj.com/site/about/guidelines.xhtml

that altering the language and/or providing epidemiologic information improved perceptions regarding LBP compared with provision of a standard report.(27, 28) This now requires confirmation in randomised trials of real patients.

The radiology report may play a role in mediating the relationship between imaging and overtreatment in LBP, (7, 29) and providing contextual information has been reported to reduce unnecessary repeat imaging, opioid prescription and specialist referral.(30, 31) Trials that have assessed the provision of epidemiologic data or additional educational messaging, (32-36) including for LBP, (20, 36, 37) have been designed to improve referrer knowledge and behaviour, but none have assessed patient knowledge or other outcomes. Our study provides evidence that commonly identified and reported terms in radiology reports are also associated with misplaced patient concerns about their implications, which may also influence their healthcare decision-making.

Those with current LBP had poorer back beliefs compared to those without a history of LBP or past LBP. This is consistent with previous studies that have found greater pessimism regarding recovery among those with current symptoms.(26, 38-41) This may also explain their increased expectation and wish for further tests and treatment.(42)

Strengths and limitations

To our knowledge this is the first study to measure societal self-reported understanding of commonly used terms in lumbar spine imaging reports and their relationship to other factors such as perceived seriousness, persistence of pain and fear of movement. Although we were unable to calculate a response rate, it is likely our results are generalisable to the five English-speaking countries we included as we used age and gender quotas to reflect the make-up of

For peer review only - http://bmjopen.bmj.com/site/about/guidelines.xhtml

each country. Additionally, the proportion of participants with a life-time history of LBP is consistent with other population-based studies, (38, 40), although the prevalence of current low back pain was slightly higher which may indicate greater interest in completing a survey about LBP. BBQ scores of our participants were also similar to other population-based cohorts.(38, 40, 41, 43, 44)

On the other hand, we used a survey sampling company, which may have introduced participation bias as participants receive rewards albeit very small ones. In addition, people who are online and familiar with computers and online questionnaires may differ from the general population. Our sample included a small number of health professionals with diverse roles and we therefore think that excluding them from our analysis would have been unlikely to change our results. We also did not consider that any meaningful conclusions could be drawn from analysing this group separately. Our study was appropriately powered and randomisation of the terms for each participant minimised any order effect bias. Where possible we adapted our survey items from other tools with established validity and reliability and the survey was piloted prior to use.

Survey measurement of public understanding of medical terms is challenging. Asking respondents whether they would need to look the term up to know what it means could be considered an even more indirect measure of self-reported understanding. As well, self-reported understanding of a medical term may not be a true reflection of participants' true understanding of terms and our study may have over or underestimated true understanding. Similarly, we did not include a measure of health literacy in our survey and we therefore do not know how differing health literacy skills may have influenced our findings. A large proportion of answers relating to the items were neutral (i.e., neither agree nor disagree),

For peer review only - http://bmjopen.bmj.com/site/about/guidelines.xhtml

however only 19 (2.8%) of participants provided neutral answers for all items and they had a wide range of BBQ scores ranging from 15-45 suggesting that their neutral answers were likely genuine.

We also performed post-hoc sensitivity analyses to determine whether our dichotomisation was an appropriate method of handling the data, and in particular the neutral responses. The association between greater self-reported understanding of the terms in people with higher education, more positive back beliefs and current LBP, as well as the lesser concern about seriousness, pain persistence and being fearful of movement among people with more positive back beliefs indicates our findings were generally robust to altering how we handled neutral responses. The sensitivity analysis did however reduce the significance of some findings, notably the relationship between education and concern about terms, indicating that some of the results of the negative binomial regression analysis should be interpreted with caution.

All of the imaging findings we assessed frequently exist in the asymptomatic population and therefore may or may not have relevance in an individual case. However, some findings such as disc bulge, disc extrusion, Modic changes, disc protrusion and disc degeneration, occur more commonly in symptomatic populations.(19) Participants' past experiences with LBP and/or imaging of the lumbar spine and these terms may have influenced their responses.

Implications for practice

All terms we investigated elicited concerns that were likely unwarranted. Combined with our finding that most participants would like access to their reports, more consideration is needed about how to report findings in lumbar imaging reports in people with LBP. Our finding that

For peer review only - http://bmjopen.bmj.com/site/about/guidelines.xhtml

respondents wanted reports to be understandable to the lay person is also consistent with the results of other studies.(45, 46)

Although clinicians may have reservations about allowing patients direct access to their medical information,(47) there is already increased availability and use of patient portals (48) and in many health systems this is standard practice.(49) In organisations where this has occurred, radiology reports are one of the most common items reviewed by patients accessing their information. (50) A recent addition to patient portals has been a lay-language glossary (51) however with a current focus on defining anatomical structures it may not reduce patient concern regarding benign findings. Any changes to patient interpretation of radiology reports as a result of using the glossary have not been evaluated.

Moreover, there is clear evidence that the low back pain beliefs and attitudes of health care professionals have an impact on patient beliefs and affect their clinical care. (52) A recent study found that the way lumbar spine imaging is reported can influence the beliefs of clinicians regarding the severity of the condition and their approach to management, including their views on whether surgery may be required. (53) Along with more in depth exploration of the impact of patient attitudes and beliefs regarding radiology findings, identifying and addressing the clinician factors that trigger low value care based upon imaging findings are important areas for future research.

It is likely that our results may be generalisable to other regional musculoskeletal conditions where imaging also often reveals age-related changes that are common in asymptomatic people. These changes include disc bulges in the cervical spine, (54) meniscal tears in the

For peer review only - http://bmjopen.bmj.com/site/about/guidelines.xhtml

knee (55) and supraspinatus tendinosis in the shoulder. (56) Our study could be replicated for these conditions to determine if our study findings are also applicable to them.

There is now both imperative and empirical data indicating the importance of accurately and clearly portraying the significance of imaging findings in terms that are understandable to both clinicians and patients.

CONCLUSION

Common and usually non-serious terms in lumbar spine imaging reports are poorly understood by the general population and may contribute to the burden of LBP. Incorporating clear explanations about the implications of these findings may reduce unwarranted anxiety and reduce low-value care.

For peer review only

Contributorship statement

CF contributed to the conception and design of this study, the acquisition, analysis and interpretation of the data, drafting and revising the work. DO contributed to the design of this study, the acquisition, analysis and interpretation of the data, drafting and revising the work. HL contributed to the conception and design of this study, the analysis and interpretation of the data and revising the work critically for content. KM contributed to the conception and design of this study, the interpretation of the data and revising the work critically for content. CM contributed to the conception and design of this study, the analysis and interpretation of the data and revising the work critically for content. DN contributed to the conception and design of this study, the interpretation of the data and revising the work critically for content. AC contributed to the conception and design of this study, the interpretation of the data and revising the work critically for content. DB contributed to the conception and design of this study, the interpretation of the data and revising the work critically for content. JJ contributed to the conception and design of this study, the interpretation of the data and revising the work critically for content. RB contributed to the conception and design of this study, the acquisition, analysis and interpretation of the data, drafting and revising the work. All authors approved the final version and agree to be accountable for all aspects of this study.

Acknowledgement

We acknowledge and thank Alex Gorelik for her assistance with data analysis for this study.

Competing interests statement

No competing interests relating to this publication have been declared. CM and RB have received grants from numerous government and not-for-profit agencies; their expenses have been covered by professional associations hosting conferences where they were speakers, and they were both investigators for a trial that received heat wraps from Flexeze at no cost.

For peer review only

Funding statement

DO is supported by an Australian National Health and Medical Research Council (NHMRC) Translating Research into Practice Fellowship (APP1168749). HL is funded by an NHMRC (grant no. APP1126767); receives project funding from the Berkeley Initiative for Transparency in the Social Sciences, a program of the Center for Effective Global Action (CEGA), with support from the Laura and John Arnold Foundation. He is also supported by funding from the National Institute for Health Research (NIHR) Collaboration for Leadership in Applied Health Research and Care Oxford at Oxford Health NHS Foundation Trust. JGJ is supported by funding from the US National Institute of Health/NIAMS (P30AR072572). CM is supported by an NHMRC Principal Research Fellowship (APP1103022). RB is supported by an NHMRC Senior Principal Research Fellowship (APP1082138).

Data sharing statement

The study protocol and deidentified participant data is available upon reasonable request to the corresponding author (caitlin.farmer1@monash.edu).

Ethical statement

This project was approved by the Monash University Human Research Ethics Committee (ID 13896). Completion of the survey was considered consent to participate.

Figure legend/caption

Figure 1: Responses to questions regarding level of understanding, seriousness, persistence of pain and fear of movement for the 14 radiology terms

For peer review only

Note: For each term, we asked four questions with a common opening ('If this term was in my report I would...') to evaluate a) understanding ('....need to look this term up to know what it means'); b) worry about seriousness ('...be worried there is a serious problem with my back'); c) worry about persistence ('...be worried my pain would persist') and d) fear of movement ('...be afraid to move in case I did more damage').

For peer review only

References

1. Hartvigsen J, Hancock MJ, Kongsted A, et al. What low back pain is and why we need to pay attention. *The Lancet*. 2018. doi:10.1016/s0140-6736(18)30480-x

2. Maher C, Underwood M, Buchbinder R. Non-specific low back pain. *The Lancet*. 2017;389(10070):736-47. doi:10.1016/s0140-6736(16)30970-9

3. National Institute for Health and Care Excellence: Clinical Guidelines. Low Back Pain and Sciatica in Over 16s: Assessment and Management. London: National Institute for Health and Care Excellence (UK); 2016.

4. Chou R, Qaseem A, Snow V, et al. Diagnosis and treatment of low back pain: A joint clinical practice guideline from the american college of physicians and the american pain society. *Annals of Internal Medicine*. 2007;147(7):478-91. doi:10.7326/0003-4819-147-7-200710020-00006

5. Oliveira CB, Maher CG, Pinto RZ, et al. Clinical practice guidelines for the management of non-specific low back pain in primary care: an updated overview. *European Spine Journal*. 2018;27(11):2791-803. doi:10.1007/s00586-018-5673-2

6. Brinjikji W, Luetmer PH, Comstock B, et al. Systematic literature review of imaging features of spinal degeneration in asymptomatic populations. *AJNR Am J Neuroradiol*. 2015;36(4):811-6. doi:10.3174/ajnr.A4173

7. Webster BS, Choi Y, Bauer AZ, et al. The cascade of medical services and associated longitudinal costs due to nonadherent magnetic resonance imaging for low back pain. *Spine (Phila Pa 1976)*. 2014;39(17):1433-40. doi:10.1097/BRS.0000000000000408

8. Webster BS, Bauer AZ, Choi Y, et al. Iatrogenic consequences of early magnetic resonance imaging in acute, work-related, disabling low back pain. *Spine (Phila Pa 1976)*. 2013;38(22):1939-46. doi:10.1097/BRS.0b013e3182a42eb6

9. McCaffery K, Nickel B, Moynihan R, et al. How different terminology for ductal carcinoma in situ impacts women's concern and treatment preferences: a randomised comparison within a national community survey. *BMJ Open*. 2015;5(11):e008094. doi:10.1136/bmjopen-2015-008094

For peer review only

For peer review only - http://bmjopen.bmj.com/site/about/guidelines.xhtml

10. Copp T, McCaffery K, Azizi L, et al. Influence of the disease label 'polycystic ovary syndrome' on intention to have an ultrasound and psychosocial outcomes: a randomised online study in young women. *Hum Reprod*. 2017;32(4):876-84. doi:10.1093/humrep/dex029

11. Scherer LD, Finan C, Simancek D, et al. Effect of "Pink Eye" Label on Parents' Intent to Use Antibiotics and Perceived Contagiousness. *Clin Pediatr (Phila)*. 2016;55(6):543-8. doi:10.1177/0009922815601983

12. Scherer LD, Zikmund-Fisher BJ, Fagerlin A, et al. Influence of "GERD" label on parents' decision to medicate infants. *Pediatrics*. 2013;131(5):839-45. doi:10.1542/peds.2012-3070

13. Scherer AM, Knaus M, Zikmund-Fisher BJ, et al. Effects of Influenza Strain Label on Worry and Behavioral Intentions. *Emerg Infect Dis*. 2017;23(8):1425-6. doi:10.3201/eid2308.170364

14. Azam N, Harrison M. Patients' perspectives on injuries. *Emerg Med J*. 2011;28(7):601-3. doi:10.1136/emj.2009.082032

15. Nickel B, Barratt A, Copp T, et al. Words do matter: a systematic review on how different terminology for the same condition influences management preferences. *BMJ Open*. 2017;7(7):e014129. doi:10.1136/bmjopen-2016-014129

16. Rosenkrantz AB. Differences in Perceptions Among Radiologists, Referring Physicians, and Patients Regarding Language for Incidental Findings Reporting. *AJR Am J Roentgenol*. 2017;208(1):140-3. doi:10.2214/AJR.16.16633

17. Bossen JK, Hageman MG, King JD, et al. Does rewording MRI reports improve patient understanding and emotional response to a clinical report? *Clin Orthop Relat Res*. 2013;471(11):3637-44. doi:10.1007/s11999-013-3100-x

18. Eysenbach G. Improving the quality of Web surveys: the Checklist for Reporting Results of Internet E-Surveys (CHERRIES). *Journal of medical Internet research*. 2004;6(3):e34-e. doi:10.2196/jmir.6.3.e34

19. Brinjikji W, Diehn FE, Jarvik JG, et al. MRI Findings of Disc Degeneration are More Prevalent in Adults with Low Back Pain than in Asymptomatic Controls: A Systematic Review and Meta-Analysis. *AJNR Am J Neuroradiol*. 2015;36(12):2394-9. doi:10.3174/ajnr.A4498

For peer review only

20. Jarvik JG, Comstock BA, James KT, et al. Lumbar Imaging With Reporting Of Epidemiology (LIRE)--Protocol for a pragmatic cluster randomized trial. *Contemp Clin Trials*. 2015;45(Pt B):157-63. doi:10.1016/j.cct.2015.10.003

21. Linton SJ, Boersma K. Early Identification of Patients at Risk of Developing a Persistent Back Problem: The Predictive Validity of The Örebro Musculoskeletal Pain Questionnaire. *The Clinical Journal of Pain*. 2003;19(2):80-6.

22. Darlow B, Perry M, Mathieson F, et al. The development and exploratory analysis of the Back Pain Attitudes Questionnaire (Back-PAQ). *BMJ Open*. 2014;4(5).

23. Robinson HS, Dagfinrud H. Reliability and screening ability of the StarT Back screening tool in patients with low back pain in physiotherapy practice, a cohort study. *BMC Musculoskeletal Disorders*. 2017;18:232. doi:10.1186/s12891-017-1553-x

24. Hill JC, Whitehurst DGT, Lewis M, et al. Comparison of stratified primary care management for low back pain with current best practice (STarT Back): a randomised controlled trial. *The Lancet*. 2011;378(9802):1560-71. doi:10.1016/s0140-6736(11)60937-9

25. Sullivan MJ, Bishop SR. The Pain Catastrophizing Scale: Development and Validation.

26. Bostick GP, Schopflocher D, Gross DP. Validity evidence for the back beliefs questionnaire in the general population. *Eur J Pain*. 2013;17(7):1074-81. doi:10.1002/j.1532-2149.2012.00275.x

27. Karran EL, Medalian Y, Hillier SL, et al. The impact of choosing words carefully: an online investigation into imaging reporting strategies and best practice care for low back pain. *PeerJ*. 2017;5:e4151. doi:10.7717/peerj.4151

28. Medalian Y, Moseley GL, Karran EL. An online investigation into the impact of adding epidemiological information to imaging reports for low back pain. *Scandinavian Journal of Pain*. 2019;19(3):629. doi:https://doi.org/10.1515/sjpain-2019-0023

29. Lemmers GPG, van Lankveld W, Westert GP, et al. Imaging versus no imaging for low back pain: a systematic review, measuring costs, healthcare utilization and absence from work. *Eur Spine J*. 2019. doi:10.1007/s00586-019-05918-1

For peer review only

30. Fried JG, Andrew AS, Ring NY, et al. Changes in Primary Care Health Care Utilization after Inclusion of Epidemiologic Data in Lumbar Spine MR Imaging Reports for Uncomplicated Low Back Pain. *Radiology*. 2018;287(2):563-9. doi:10.1148/radiol.2017170722

31. McCullough BJ, Johnson GR, Martin BI, et al. Lumbar MR imaging and reporting epidemiology: do epidemiologic data in reports affect clinical management? *Radiology*. 2012;262(3):941-6. doi:10.1148/radiol.11110618

32. Heidenreich PA, Gholami P, Sahay A, et al. Clinical reminders attached to echocardiography reports of patients with reduced left ventricular ejection fraction increase use of beta-blockers: a randomized trial. *Circulation*. 2007;115(22):2829-34. doi:10.1161/CIRCULATIONAHA.106.684753

33. Heidenreich PA, Chacko M, Goldstein MK, et al. ACE inhibitor reminders attached to echocardiography reports of patients with reduced left ventricular ejection fraction. *Am J Med*. 2005;118(9):1034-7. doi:10.1016/j.amjmed.2004.12.028

34. Stock JL, Wand CE, Coderre JA, et al. Clinical Reporting to Primary Care Physicians Leads to Increased Use and Understanding of Bone Densitometry and Affects the Management of Osteoporosis. *Annals of Internal Medicine*. 1998;128(12_Part_1). doi:10.7326/0003-4819-128-12_Part_1-199806150-00006

35. Ramsay CR, Eccles M, Grimshaw JM, et al. Assessing the long-term effect of educational reminder messages on primary care radiology referrals. *Clinical Radiology*. 2003;58(4):319-21.

36. Eccles M, Steen N, Grimshaw J, et al. Effect of audit and feedback, and reminder messages on primary-care radiology referrals: a randomised trial. *Lancet*. 2001;357(9266):1406-9.

37. Jarvik JG, Meier EN, James KT, et al. The Effect of Including Benchmark Prevalence Data of Common Imaging Findings in Spine Image Reports on Health Care Utilization Among Adults Undergoing Spine Imaging: A Stepped-Wedge Randomized Clinical Trial. *JAMA Network Open*. 2020;3(9):e2015713-e. doi:10.1001/jamanetworkopen.2020.15713

38. Gross DP, Ferrari R, Russell AS, et al. A population-based survey of back pain beliefs in Canada. *Spine (Phila Pa 1976)*. 2006;31(18):2142-5. doi:10.1097/01.brs.0000231771.14965.e4

39. Darlow B, Perry M, Stanley J, et al. Cross-sectional survey of attitudes and beliefs about back pain in New Zealand. *BMJ Open*. 2014;4(5):e004725. doi:10.1136/bmjopen-2013-004725

For peer review only

40. Buchbinder R, Jolley D. Effects of a media campaign on back beliefs is sustained 3 years after its cessation. *Spine (Phila Pa 1976)*. 2005;30(11):1323-30. doi:10.1097/01.brs.0000164121.77862.4b

41. Buchbinder R, Jolley D, Wyatt M. Population based intervention to change back pain beliefs and disability: three part evaluation. *BMJ*. 2001;322(7301):1516-20. doi:10.1136/bmj.322.7301.1516

42. Jenkins HJ, Hancock MJ, Maher CG, et al. Understanding patient beliefs regarding the use of imaging in the management of low back pain. *Eur J Pain*. 2016;20(4):573-80. doi:10.1002/ejp.764

43. Buchbinder R, Jolley D. Population based intervention to change back pain beliefs: three year follow up population survey. *BMJ*. 2004;328(7435):321. doi:10.1136/bmj.328.7435.321

44. Gross DP, Russell AS, Ferrari R, et al. Evaluation of a Canadian Back Pain Mass Media Campaign. *Spine*. 2010;35(8):906-13. doi:10.1097/BRS.0b013e3181c91140

45. Cabarrus M, Naeger DM, Rybkin A, et al. Patients Prefer Results From the Ordering Provider and Access to Their Radiology Reports. *J Am Coll Radiol*. 2015;12(6):556-62. doi:10.1016/j.jacr.2014.12.009

46. Johnson AJ, Easterling D, Williams LS, et al. Insight from patients for radiologists: improving our reporting systems. *J Am Coll Radiol*. 2009;6(11):786-94. doi:10.1016/j.jacr.2009.07.010

47. Johnson AJ, Frankel RM, Williams LS, et al. Patient Access to Radiology Reports: What Do Physicians Think? *J*. 2010;7(4):281-9. doi:http://dx.doi.org/10.1016/j.jacr.2009.10.011

48. Henshaw D, Okawa G, Ching K, et al. Access to radiology reports via an online patient portal: Experiences of referring physicians and patients. *J*. 2015;12(6):582-6.e1. doi:http://dx.doi.org/10.1016/j.jacr.2015.01.015

49. Zanaboni P, Ngangue P, Mbemba GIC, et al. Methods to Evaluate the Effects of Internet-Based Digital Health Interventions for Citizens: Systematic Review of Reviews. *J Med Internet Res*. 2018;20(6):e10202. doi:10.2196/10202

50. Miles RC, Hippe DS, Elmore JG, et al. Patient Access to Online Radiology Reports: Frequency and Sociodemographic Characteristics Associated with Use. *Acad Radiol*. 2016;23(9):1162-9. doi:10.1016/j.acra.2016.05.005

51. Oh SC, Cook TS, Kahn CE, Jr. PORTER: a Prototype System for Patient-Oriented Radiology Reporting. *J Digit Imaging*. 2016;29(4):450-4. doi:10.1007/s10278-016-9864-2

For peer review only - http://bmjopen.bmj.com/site/about/guidelines.xhtml

52. Darlow B, Fullen BM, Dean S, et al. The association between health care professional attitudes and beliefs and the attitudes and beliefs, clinical management, and outcomes of patients with low back pain: a systematic review. *Eur J Pain*. 2012;16(1):3-17. doi:10.1016/j.ejpain.2011.06.006

53. Rajasekaran S, Dilip Chand Raja S, Pushpa BT, et al. The catastrophization effects of an MRI report on the patient and surgeon and the benefits of 'clinical reporting': results from an RCT and blinded trials. *Eur Spine J*. 2021. doi:10.1007/s00586-021-06809-0

54. Nakashima H, Yukawa Y, Suda K, et al. Abnormal findings on magnetic resonance images of the cervical spines in 1211 asymptomatic subjects. *Spine (Phila Pa 1976)*. 2015;40(6):392-8. doi:10.1097/BRS.0000000000000775

55. Culvenor AG, Oiestad BE, Hart HF, et al. Prevalence of knee osteoarthritis features on magnetic resonance imaging in asymptomatic uninjured adults: a systematic review and meta-analysis. *Br J Sports Med*. 2018. doi:10.1136/bjsports-2018-099257

56. Girish G, Lobo LG, Jacobson JA, et al. Ultrasound of the shoulder: asymptomatic findings in men. *AJR Am J Roentgenol*. 2011;197(4):W713-9. doi:10.2214/AJR.11.6971

For peer review only

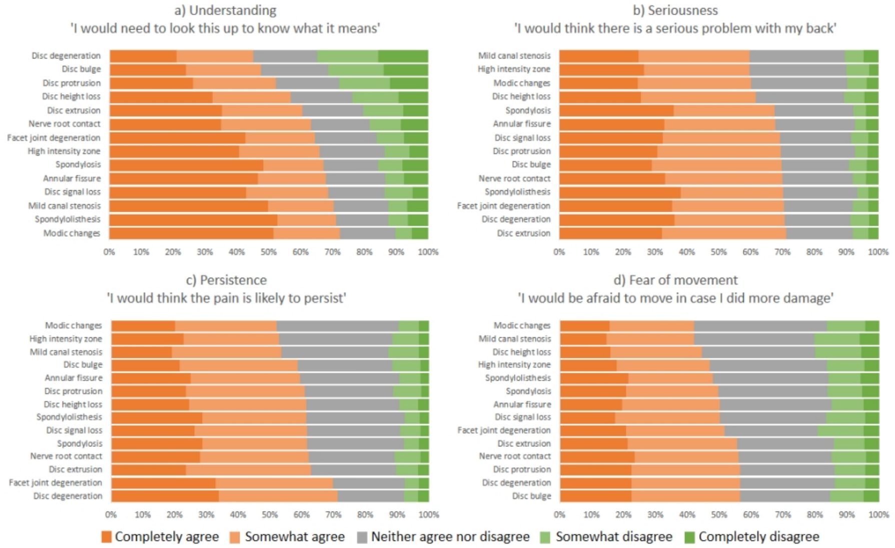

Figure 1: Responses to questions regarding level of understanding, seriousness, persistence of pain and fear of movement for the 14 radiology terms

Note: For each term, we asked four questions with a common opening ('If this term was in my report I would...') to evaluate a) understanding ('....need to look this term up to know what it means'); b) worry about seriousness ('...be worried there is a serious problem with my back'); c) worry about persistence ('...be worried my pain would persist') and d) fear of movement ('...be afraid to move in case I did more damage').

46x26mm (700 x 700 DPI)

Supplementary File

Note that for all questions the current analysis is dichotomised into strongly disagree/somewhat disagree as one value and Neutral/Somewhat agree/Strongly agree as the other.

Sensitivity analysis A: Questions regarding seriousness, persistence and fear of movement dichotomised into Strongly disagree/Somewhat disagree/neutral compared with Somewhat agree/Strongly agree. Comprehension remains unchanged.

Table 1: ***Association between the self-reported understanding of the 14 terms overall and perceived seriousness, pain persistence and fear of movement, Spearman's rho correlation****

	Understanding	Seriousness	Pain persistence	Fear of movement
**Understanding**	1			
**Seriousness**	0.29	1		
**Persistence**	0.21	0.72	1	
**Fear of movement**	0.19	0.53	0.6	1

**All p values <0.001.*

Values between 0.10 and 0.29 represent a weak association, 0.30-0.49 moderate and 0.5 and above a strong association.

For peer review only

Table 2: Association between self-reported understanding of the 14 terms, perceived seriousness, pain persistence and fear of movement and demographic details, history of back pain and back beliefs

	**Understanding**	**Seriousness**	**Pain persistence**	**Fear of movement**
	Regression coefficient (95% Confidence Interval)			
**Education (REF - High school)**				
**Trade/Diploma**	0.36 (-0.01, 0.73)	0.19 (-0.17, 0.56)	-0.07 (-0.42, 0.29)	-0.12 (-0.49, 0.24)
**Bachelor**	**0.40 (0.07, 0.72)***	0.02 (-0.28, 0.32)	0.04 (-0.26, 0.34)	-0.09 (-0.39, 0.22)
**Postgraduate qualifications**	**0.66 (0.23, 1.08)****	-0.07 (-0.46, 0.33)	-0.15 (-0.53, 0.23)	0.03 (-0.37, 0.43)
**LBP History (REF = Never)**				
**Previous LBP**	0.27 (-0.21, 0.75)	0.12 (-0.28, 0.52)	0.15 (-0.23, 0.53)	-0.06 (-0.46, 0.36)
**Current LBP**	**0.50 (0.02, 0.97)***	0.06 (-0.34, 0.47)	0.14 (-0.25, 0.52)	**-0.60 (-1.00, -0.19)****
**BBQ score**	**0.02 (0.0003, 0.04)***	**-0.02 (-0.04, -0.005)***	**-0.05 (-0.07, -0.03)*****	**-0.07 (-0.09, -0.05)*****
**Age, years**	0.002 (-0.007, 0.01)	0.002 (-0.01, 0.01)	0.003 (-0.004, 0.011)	**-0.01 (-0.02, -0.01)****
**Gender (REF = Female)**				
**Male**	0.07 (-0.20, 0.33)	**-0.39 (-0.64, -0.14)****	**-0.32 (-0.57, -0.08)***	-0.24 (-0.49, 0.01)

Note: Bold indicates statistically significant, * $p < 0.05$, ** $p < 0.01$, *** $p < 0.001$. Results rounded to two decimal points or first meaningful decimal point. REF = Reference category, LBP = Low Back Pain, BBQ = Back Beliefs Questionnaire

Continuous variables (BBQ score and age): For understanding, a positive regression coefficient indicates greater self-reported understanding is associated with better back beliefs (higher BBQ score) and increased age. For seriousness, pain persistence and fear of movement, a positive regression coefficient indicates greater worry/concern is associated with poorer back beliefs (lower BBQ score) and increased age.

Categorical variables: For understanding, a positive regression coefficient indicates increased self-reported understanding compared with the reference category. For seriousness, pain persistence and fear of movement, a positive regression coefficient indicates greater worry/concern compared with the reference variable.

For peer review only

Sensitivity analysis B: Neutral answers removed. Dichotomy is strongly disagree/somewhat disagree vs somewhat agree/strongly agree.

Table 3: ***Association between the self-reported understanding of the 14 terms overall and perceived seriousness, pain persistence and fear of movement, Spearman's rho correlation****

	Understanding	Seriousness	Pain persistence	Fear of movement
**Understanding**	1			
**Seriousness**	0.60	1		
**Persistence**	0.45	0.72	1	
**Fear of movement**	0.37	0.53	0.6	1

****All p values <0.001.***

Values between 0.10 and 0.29 represent a weak association, 0.30-0.49 moderate and 0.5 and above a strong association.

For peer review only

Table 4: Association between self-reported understanding of the 14 terms, perceived seriousness, pain persistence and fear of movement and demographic details, history of back pain and back beliefs

	**Understanding**	**Seriousness**	**Pain persistence**	**Fear of movement**
	Regression coefficient (95% Confidence Interval)			
Education (REF - High school)				
Trade/Diploma	0.27 (-0.11, 0.65)	0.005 (-0.59, 0.60)	-0.42 (-0.97, 0.13)	-0.39 (-0.88, 0.09)
Bachelor	0.32 (-0.01, 0.66)	- 0.16 (-0.63, 0.31)	-0.19 (-0.63, 0.26)	**-0.45 (-0.87, -0.04)***
Postgraduate qualifications	**0.55 (0.12, 0.98)***	0.43 (-1.03, 0.17)	**-0.77 (-1.32, -0.23)****	**-0.65 (-1.15, -0.14)***
LBP History (REF = Never)				
Previous LBP	0.18 (-0.30, 0.66)	0.21 (-0.41, 0.83)	-0.01 (-0.58, 0.56)	-0.19 (-0.71, 0.33)
Current LBP	**0.55 (0.07, 1.02)***	-0.01 (-0.63, 0.61)	-0.18 (-0.76, 0.40)	**-0.61 (-1.14, -0.08)***
BBQ score	**0.03 (0.01, 0.05)***	**-0.04 (-0.07, -0.01)****	**-0.08 (-0.11, -0.05)*****	**-0.08 (-0.11, -0.05)*****
Age, years	-0.003 (-0.01, 0.01)	**0.02 (0.01, 0.04)*****	**0.03 (0.02, 0.04)*****	-0.01 (-0.02, 0.001)
Gender (REF = Female)				
Male	0.17 (-0.10, 0.44)	**-0.49 (-0.90, -0.08)***	-0.27 (-0.64, 0.11)	-0.30 (-0.64, 0.04)

Note: Bold indicates statistically significant, * p < 0.05, **p < 0.01, ***p < 0.001. Results rounded to two decimal points or first meaningful decimal point. REF = Reference category, LBP = Low Back Pain, BBQ = Back Beliefs Questionnaire

Continuous variables (BBQ score and age): For understanding, a positive regression coefficient indicates greater self-reported understanding is associated with better back beliefs (higher BBQ score) and increased age. For seriousness, pain persistence and fear of movement, a positive regression coefficient indicates greater worry/concern is associated with poorer back beliefs (lower BBQ score) and increased age.

Categorical variables: For understanding, a positive regression coefficient indicates increased self-reported understanding compared with the reference category. For seriousness, pain persistence and fear of movement, a positive regression coefficient indicates greater worry/concern compared with the reference variable.

For peer review only

Cherries checklist – Consumer understanding of terms used in imaging reports requested for low back pain

	Category	Checklist item	Page(s)
1	Design	**Describe survey design**<br>Describe target population, sample frame. Is the sample a convenience sample? (In 'open' surveys this is most likely).	6-11
2	IRB (Institutional Review Board) approval and informed consent process	**IRB approval**<br>Mention whether the study has been approved by an IRB.	10
3		**Informed consent**<br>Describe the informed consent process. Where were the participants told the length of time of the survey, which data were stored and where and for how long, who the investigator was, and the purpose of the study?	10
4		**Data protection**<br>If any personal information was collected or stored, describe what mechanisms were used to protect unauthorised access.	N/A
5	Development and pre-testing	**Development and testing**<br>Stated how the survey was developed, including whether the usability and technical functionality of the electronic questionnaire had been tested before fielding the questionnaire	7
6	Recruitment process and description of the sample having access to the questionnaire	**Open survey vs closed survey**<br>An 'open survey' is a survey open for each visitor of a site, while a closed survey is only open to a sample which the investigator knows (password-protected survey).	6-7
7		**Contact mode**<br>Indicate whether or not the initial contact with the potential participants was made on the internet (Investigators may also send out questionnaires by mail and allow for Web-based data entry).	6-7
8		**Advertising the survey**<br>How/where was the survey announced or advertised? Some examples are offline media (newspapers), or online (mailing lists – if yes, which ones?) or banner ads (where were these banner ads posted and what did they look like?). It is important to know the wording of the announcement as it will heavily influence who chooses to participate. Ideally the survey announcement should be published as an appendix.	6-7
9	Survey administration	**Web/email**<br>State the type of e-survey (eg one posted on a website, or one sent out through email). If it is an email survey, were the responses entered manually into a database, or was there an automatic method for capturing responses?	6
10		**Context**<br>Describe the website (for mailing list/newsgroup) in which the survey was posted. What is the website about, who is visiting it, what are visitors normally looking for? Discuss to what degree the content of the website could pre-select	N/A

For peer review only

		the sample or influence the result. For example, a survey about vaccination on an anti-immunisation website will have different results from a web survey conducted on a N/A government website.	
11		**Mandatory/voluntary**<br>Was it a mandatory survey to be filled in by every visitor who wanted to enter the website, or was it a voluntary survey?	N/A
12		**Incentives**<br>Were any incentives offered (eg monetary, prizes, or non-monetary incentives such as an offer to provide the survey results)?	6-7
13		**Time/Date**<br>In what timeframe were the data collected?	6
14		**Randomisation of items or questionnaires**<br>To prevent biases items can be randomised or alternated	7
15		**Adaptive questioning**<br>Use adaptive questioning (certain items, or only conditionally displayed based on responses to other items) to reduce number and complexity of the questions	6-7
16		**Number of items**<br>What was the number of questionnaire items per page? The number of items is an important factor for the completion rate.	7-8
17		**Number of screens (pages)**<br>Over how many pages was the questionnaire distributed? The number of items is an important factor for the completion rate.	N/A
18		**Completeness check**<br>It is technically possible to do consistency or completeness checks before the questionnaire is submitted. Was this done, and if 'yes' how (usually JAVAscript)? An alternative is to check for completeness after the questionnaire has been submitted (and highlight mandatory items). If this has been done, it should be reported. All items should provide a non-response option such as 'not applicable' or 'rather not say' and selection of one response option should be enforced.	9
19		**Review step**<br>State whether respondents were able to review and change their answers (eg through a Back button or a Review step which displays a summary of the responses and asks the respondents if they are correct).	10
20	Response rates	**Unique site visitor**<br>If you provide view rates or participation rates, you need to define how you determined a unique visitor. There are different techniques available, based on IP addresses or cookies or both.	N/A

For peer review only

21		**View rate**<br>**(ratio of unique survey visitors/unique site visitors)**<br>Requires counting unique visitors to the first page of the survey, divided by the number of unique site visitors (not page views!). It is not unusual to have view rates of less than 0.1% if the survey is voluntary.	N/A
22		**Participation rate**<br>**(Ratio of unique visitors who agreed to participate/unique first survey page visitors)**<br>Count the unique number of people who filled in the first survey page (or agreed to participate, for example by checking a checkbox), divided by visitors who visit the first page of the survey (or the informed consents page, if present). This can also be called 'recruitment' rate.	N/A
23		**Completion rate**<br>**(Ratio of users who finished the survey/users who agreed to participate)**<br>The number of people submitting the last questionnaire page, divided by the number of people who agreed to participate (or submitted the first survey page). This is only relevant if there is a separate 'informed consent' page or if the survey goes over several pages. This is a measure for attrition. Note that 'completion' can involve leaving questionnaire items blank. This is not a measure for how completely questionnaires were filled in. (If you need a measure for this use the word 'completeness rate'.)	11
24	Preventing multiple entries from the same individual	**Cookies used**<br>Indicate whether cookies were used to assign a unique user identifier to each client computer. If so, mention the page on which the cookie was set and read, and how long the cookie was valid. Were duplicate entries avoided by preventing users access to the survey twice; or were duplicate database entries having the same user ID eliminated before analysis? If the latter, which entries were kept for analysis (eg the first entry or the most recent)?	N/A
25		**IP check**<br>Indicate whether the IP address of the client computer was used to identify potential duplicate entries from the same user. If so, mention the period of time for which no two entries from the same IP address were allowed (eg 24 hours). Were duplicate entries avoided by preventing users with the same IP address access to the survey twice; or were duplicate database entries having the same IP address within a given period of time eliminated before analysis? If the latter, which entries were kept for analysis (eg the first entry or the most recent)?	9
26		**Log file analysis**<br>Indicate whether other techniques to analyse the log file for identification of multiple entries were used. If so, please describe.	N/A
27		**Registration**<br>In 'closed' (non-open) surveys, users need to login first and it is easier to prevent duplicate entries from the same user. Describe how this was done. For example, was the survey never displayed a second time once the user had filled	N/A

For peer review only

		it in, or was the username stored together with the survey results and later eliminated? If the latter, which entries were kept for analysis (eg the first entry or the most recent)?	
28	Analysis	**Handling of incomplete questionnaires**<br>Were only completed questionnaires analysed? Were questionnaires which terminated early (where, for example, users did not go through all questionnaire pages) also analysed?	9, 11
29		**Questionnaires submitted with an atypical timestamp**<br>Some investigators may measure the time people needed to fill in a questionnaire and exclude questionnaires that were submitted too soon. Specify the timeframe that was used as a cut-off point and describe how this point was determined.	N/A
30		**Statistical correction**<br>Indicate whether any methods such as weighting of items or propensity scores have been used to adjust for the non-representative sample; if so, please describe the methods.	N/A

For peer review only
